# High Precision Outdoor and Indoor Reference State Estimation for Testing Autonomous Vehicles †

**DOI:** 10.3390/s21041131

**Published:** 2021-02-06

**Authors:** Eduardo Sánchez Morales, Julian Dauth, Bertold Huber, Andrés García Higuera, Michael Botsch

**Affiliations:** 1 Technische Hochschule Ingolstadt, Esplanade 10, 85049 Ingolstadt, Germany; j-dauth@gmx.de (J.D.); michael.botsch@thi.de (M.B.); 2 GeneSys Elektronik GmbH, In der Spöck 10, 77656 Offenburg, Germany; Huber@genesys-offenburg.de; 3 European Parliamentary Research Service, Rue Wiertz 60, B-1047 Brussels, Belgium; andres.garcia@europarl.europa.eu

**Keywords:** machine learning, autonomous vehicles, Inertial Navigation System, Satellite Navigation, Real-Time Kinematic, indoor navigation, reference state

## Abstract

A current trend in automotive research is autonomous driving. For the proper testing and validation of automated driving functions a reference vehicle state is required. Global Navigation Satellite Systems (GNSS) are useful in the automation of the vehicles because of their practicality and accuracy. However, there are situations where the satellite signal is absent or unusable. This research work presents a methodology that addresses those situations, thus largely reducing the dependency of Inertial Navigation Systems (INSs) on the SatNav. The proposed methodology includes (1) a standstill recognition based on machine learning, (2) a detailed mathematical description of the horizontation of inertial measurements, (3) sensor fusion by means of statistical filtering, (4) an outlier detection for correction data, (5) a drift detector, and (6) a novel LiDAR-based Positioning Method (LbPM) for indoor navigation. The robustness and accuracy of the methodology are validated with a state-of-the-art INS with Real-Time Kinematic (RTK) correction data. The results obtained show a great improvement in the accuracy of vehicle state estimation under adverse driving conditions, such as when the correction data is corrupted, when there are extended periods with no correction data and in the case of drifting. The proposed LbPM method achieves an accuracy closely resembling that of a system with RTK.

## 1. Introduction

Autonomous driving has become a popular trend in automotive research. The motivation for autonomous driving ranges from comfort or practicality functions to safety critical applications. Independently of the final use, the fact that the vehicles are machines that can cause serious harm to humans in the event of a malfunction has to be taken into consideration. Therefore, autonomous driving functions need to be subjected to an extensive process of testing and validation which requires a highly accurate reference vehicle state.

A common practice to generate such a highly accurate reference vehicle state is the fusion of data from Inertial Measurement Units (IMU) with measurements from external sensors. Given the extensive scientific community working on these systems, their practicality, and especially their high accuracy, Satellite Navigation (SatNav) receivers have become the most common choice of sensor to fuse with IMUs. Even consumer-grade receivers are capable of acquiring information from various GNSS (such as GPS, GLONASS, Galileo, Beidou, etc.), which enables the receivers to accurately estimate several state variables. Unfortunately, there are common driving situations where satellite signals are either absent or corrupted to an extent that renders them unusable. Some examples of these driving situations are tunnels, bridge underpasses, parking structures or testing halls.

The objective of the present work is to reduce the dependency of INSs on external sensors, while still being able to generate a reference vehicle state. Six key aspects of the estimation of the vehicle state are addressed: (i) the standstill recognition (Section 2.1), (ii) the horizontation of IMU measurements (Section 2.2), (iii) sensor fusion (Section 2.3), (iv) drift detection (Section 2.4), (v) outlier detection for correction data (Section 2.5), and (vi) highly accurate indoor navigation (Section 2.6). It will be shown that the proposed methods are able to provide important improvements in the estimation of the vehicle state even when used individually. Furthermore, if used in combination, the proposed methods allow for an accurate enough estimation of the vehicle state to test and validate autonomous driving functions even in indoor environments.

For an adequate estimation of the vehicle state by means of IMUs, it is essential to ascertain when the vehicle is not moving because it is at this time that both the velocity over ground and the proper acceleration are equal to zero [1]. Even though, historically, vehicle speed sensors were designed to measure velocity rather than when a vehicle is at a standstill [2], they are now able to accurately measure a standstill. Nonetheless, there could be disadvantages if external devices continuously read the vehicle sensor data in real time (for example, through the OBDII interface). Even when various methods [3,4,5] and tools used by manufacturers [6] theoretically allow a CAN-bus use of ≈80% [7], recent studies suggest that CAN-bus messages can still miss their deadlines [8,9,10]. The traffic within a CAN-bus would increment if an external device continuously accesses the vehicle sensor data in real time, which could further increase the risk that the CAN-bus messages miss their deadlines. Consequently, more research is required before considering fusing the vehicle on-board sensors with external IMUs. Alternative sensors for measuring the velocity over ground, such as the Wheel Pulse Transducer (WPT) [11] or the Correvit [12,13] increase testing costs and complexity.

To the best of authors’ knowledge, the only method specifically designed for standstill detection is patented by Robert Bosch GmbH [14]. This method compares the position of an object in consecutive frames of a video provided by a camera in the vehicle, and an algorithm estimates the velocity of the vehicle relative to the object. As with all camera-based methods, there is a trade-off between the spatial resolution and the distance to the recorded objects, which negatively affects the standstill recognition.

The method proposed in this work for standstill recognition is based on a machine learning classifier, and has the key advantage that all required inputs are generated solely with IMU measurements. No additional sensors or infrastructural elements are required, which reduces testing complexity, improves the robustness of the method and makes it highly adequate for indoor navigation.

The state of the vehicle can be estimated once the algorithm has reliably established that the vehicle is moving. If Gauss-Markov stochastic processes are assumed, the most computationally efficient method to estimate the *optimum* state of moving objects is the Kalman Filter (KF) [15]. The KF algorithm can fuse sensor measurements with the vehicle state predicted by means of a motion model. Since the aim of this work is to generate a reference vehicle state for testing and validation of autonomous driving functions, the motion model used is especially designed for accurate performance under tractive driving.

Two critical parameters for the KF are the measurement noise and the system noise, because they are required to calculate the Kalman gain. Therefore, two methods are proposed to monitor both values, and thus to improve the robustness of the KF: a drift recognition and an outlier detector.

The drift recognition tackles the task of monitoring the vehicle state to detect when the vehicle is drifting. This information is extremely valuable because the motion model used in this research work is optimized for tractive-driving situations. When the vehicle starts to drift, the previously estimated system noise is no longer valid. Knowing when the vehicle is drifting enables the possibility of adjusting the system noise or switching to a better suited motion model.

Other works found in the literature address the issue of drifting too, however, they tend to limit the scope of their research to simulation environments [16,17] or require additional parameters that cannot easily be estimated with an IMU [18]. Conversely, the drift recognition in this work is designed to operate by performing consistency checks between two estimated state variables: the lateral acceleration and the sideslip angle. As demonstrated later, these state variables provide enough information to perform a robust drift detection.

The outlier detector serves as a complement to the drift detector. Although the drift detector monitors the validity of the motion model, the outlier detector does the same for the measurements. This is especially beneficial for SatNav measurements due to the multi-path effect. This effect appears when the signal received from the satellites does not follow a straight path, but bounces off other objects before reaching the SatNav receiver. The multi-path effect can occur as the vehicle drives near objects that are able to reflect the SatNav signal, such as trailers, tall buildings or metallic structures, and causes problems for the methods that measure the vehicle state (trilateration, carrier-phase measurements, etc.). A relevant consequence of the multi-path effect is that it reduces the accuracy of the measured vehicle state. It is quite common to see significant jumps in the measured state variables while the multi-path effect is present. There are several approaches in the literature to deal with the multi-path problem, such as [19,20,21,22,23]. However, to implement these approaches would require access to the pseudo-ranges of the SatNav receivers which is not possible for most consumer-grade devices. Therefore, the problem is addressed by filtering outliers. This filtering is achieved by a consistency check of the measurement vector before it is used as correction data.

In this research work a dynamic low-pass filter is designed to refine the outlier detection method by means of taking the measurement noise into account. This dynamic low-pass filter is highly beneficial for all sensors that estimate a quality indicator for their own measurements, as is the case of the “dilution of precision”, “diminution of precision” [24,25] or standard deviation of the SatNav receivers. Many sensors can track the accuracy associated with the measurements that they perform, but are not always capable of detecting estimation errors of this self-reported accuracy.

The investments made in GNSS [26,27] have provided various advantages, such as (i) high accuracy [28,29], (ii) a low cost for end users [30], and (iii) an extensive scientific community that works on refining its measurements and overcoming its limitations [31]. Arguably, these three advantages make the GNSS the best choice for external sensors in open-sky use-cases. However, there are still situations where the GNSS are not available or usable, as is the case in tunnels, parking structures or urban canyons. Moreover, there is an interest in testing in indoor halls because this allows engineers to test sensors, algorithms and systems under simulated and reproducible weather conditions such as day, night, rain, fog or even SatNav outages.

Due to the aforementioned reasons, it is necessary to investigate approaches for acquiring feedback in closed-sky situations. Comprehensive surveys on indoor positioning systems can be found in [32,33]. These approaches are based on the combination of diverse methods and sensors, such as WiFi, sonar, radar, or proprietary technologies such as the iBeacon [34].

The LiDAR-based approach with the best performance is presented on [35] with a mean localization error of 11.5 cm and standard deviation of 5.4 cm. The accuracy is evaluated by comparing the outputs of the algorithm to “human-labeled” ground-truth data. The indoor positioning system with the best performance that was found is the “Active Bat” [36,37] with a mean localization error of 3 cm, but the evaluation methodology is not clearly specified [38]. The biggest disadvantage of the Active Bat is the numerous receivers required which must be placed with separations of 1.2 m and must cover the complete measurement area. Therefore, the installation of such a system might not be practical. Other indoor positioning systems already on the market [39,40,41] are designed for applications that do not require a centimeter-precise localization. Alternative navigation methods that use LiDARs are presented in [42,43,44]. However, the existing methods still face a series of challenges, such as the positioning accuracy, the number of state variables they can measure or the algorithm runtime.

## 2. Materials and Methods

**Figure 1:**
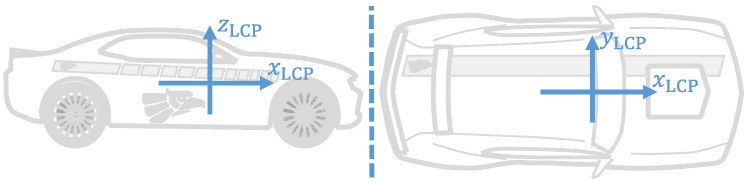
Graphical depiction of the Local Car Plane.

The present work deals with the measurements provided by different sensors, such as IMUs, LiDARs, SatNav receivers, etc. For simplification purposes, it is assumed that all sensors output their measurements in the Local Car Plane (LCP). The LCP is the vehicle reference frame and is defined analogue to the ISO 8855:2011 norm [45]. The LCP is composed by the mutually perpendicular $x_{\mathrm{LCP}}$, $y_{\mathrm{LCP}}$, and $z_{\mathrm{LCP}}$ axes, with $\vec{z_{\mathrm{LCP}}}=\vec{x_{\mathrm{LCP}}}\times \vec{y_{\mathrm{LCP}}}$ and origin $o_{\mathrm{LCP}}$ at the Center of sprung Mass (CoM) of the vehicle. The $x_{\mathrm{LCP}}$ axis is parallel to the longitudinal axis of the vehicle and points towards the hood, the $z_{\mathrm{LCP}}$ axis points upwards, and the $y_{\mathrm{LCP}}$ axis is given according to the right-hand rule. The LCP is illustrated in Figure 1.

The vehicles move on the Local Tangent Plane (LTP). The LTP is a Cartesian reference frame, composed by the mutually perpendicular $x_{\mathrm{LTP}}$, $y_{\mathrm{LTP}}$ and $z_{\mathrm{LTP}}$ axes, with $\vec{z_{\mathrm{LTP}}}=\vec{x_{\mathrm{LTP}}}\times \vec{y_{\mathrm{LTP}}}$; and with origin $o_{\mathrm{LTP}}$ at an arbitrary location on the surface of the Earth. The axis orientation of the LTP is similar to the East-North-Up (ENU) reference frame, where the $x_{\mathrm{LTP}}\times y_{\mathrm{LTP}}$-plane is perpendicular to the gravitational pull of the Earth, the $y_{\mathrm{LTP}}$ axis points to the true north of the Earth, and the $z_{\mathrm{LTP}}$ axis is parallel to the gravitational pull of the Earth, and points upwards.

### 2.1. Standstill Recognition

The first step for estimating the vehicle state is the standstill detection. The strategy to achieve this, is to generate features from the IMU signals that can describe whether the vehicle is standing still or not. The generated features are then classified by means of machine learning. The proposed method uses the Random Forest (RF) [46] for classifying standstill or motion according to the features generated from the IMU signals as inputs. The classes used are shown in Table 1. False positives are unacceptable because they can affect greatly the estimation of the vehicle state, especially if they occur at highway velocities. False negatives can be tolerated because, as shown later, their effect is negligible for the estimation of the vehicle state. If this holds, the standstill classification is considered robust.

The use of the RF is justified mainly because it is not possible to manually determine thresholds for the IMU signals that allow a robust standstill classification. This happens because some driving conditions are almost equal to a standstill, such as cruising on a highway or driving at walking velocity. This fact is further strengthened when considering the numerous configurations possible for the same vehicle chassis (engine, suspension, tires, etc.), and that even small components, such as the engine mounts, can have a significant impact on the vibrations and rotations sensed by the IMU.

The RF not only enables the implementation of a robust standstill recognition, but a 10 min dataset is also enough for a robust classification, and the training process is done within seconds. Also, given that the RF is real-time capable, it allows the online use of the proposed method. Further details about the RF can be found in [46]. The procedure to recognize a standstill consists of three steps: (i) generation of the training dataset, (ii) training of the RF, and (iii) classification of the vehicle state. The specifics applicable to the present research work is detailed below.

The first step to recognize a standstill, is to generate a training dataset for the RF. The 10 min-long dataset that is required to generate the features for the RF is composed by the proper acceleration vector $a_{\mathrm{LCP}}$ and the composition of rotations ${\dot{\theta }}_{\mathrm{LCP}}$ that are measured by the IMU, these are such that

(1) $$a_{\mathrm{LCP}}={\left[\begin{array}{ccc} a_{x,\mathrm{LCP}} & a_{y,\mathrm{LCP}} & a_{z,\mathrm{LCP}} \end{array}\right]}^{T},$$

(2) $${\dot{\theta }}_{\mathrm{LCP}}={\left[\begin{array}{ccc} {\dot{\theta }}_{\mathrm{roll}} & {\dot{\theta }}_{\mathrm{pitch}} & {\dot{\theta }}_{\mathrm{yaw}} \end{array}\right]}^{T}.$$

The training dataset for the RF is equally distributed between three conditions: standstill, moving at walking pace and random driving.

For the standstill condition, the vehicle should be as motionless as possible, but ready to drive off. In the case of vehicles with automatic transmission, “drive” should be selected; and vehicles with manual transmission should be either in neutral or in gear and with the clutch engaged. The reason for this, is to let the IMU sense engine and drivetrain vibrations as well. During the standstill, all dead loads are negligible, and all live loads are undesirable. Except for engine and drivetrain, all actions that provoke chassis vibration or motion should be avoided: passenger movement, vehicle loading, opening or closing doors, trunk or hood, etc.

For the condition of moving at walking pace, the vehicle is driven as slow as possible and on a straight line. Contact with pedals or steering wheel should be avoided. This allows one to generate samples with the “motion” label but that are very similar to a “standstill”, which is highly relevant for differentiating between both.

For the condition of random driving, the vehicle is driven with random accelerations, velocities and directions. The more diverse, the better for the dataset.

Next, the features for the RF are generated in the time and frequency domain. The features in the time domain are: (i) $a_{x,\mathrm{LCP}}^{2}+a_{y,\mathrm{LCP}}^{2}$, (ii) $a_{z,\mathrm{LCP}}^{2}$, (iii) ${\dot{\theta }}_{\mathrm{roll}}^{2}+{\dot{\theta }}_{\mathrm{pitch}}^{2}$, and (iv) ${\dot{\theta }}_{\mathrm{yaw}}^{2}$.

The features in the frequency domain are created from the features in the time domain by generating the Discrete Fourier Transform (DFT) of each feature in the time domain. In the present work, the DFT is generated with a MATLAB Toolbox that is based on [47,48,49]. The frequency range that the DFT uses is given as in Equation (3).

(3) $$\begin{array}{c} {}[0\;:\;\frac{F_{s}}{n_{\mathrm{DFT}}}\;:\;F_{s}], \end{array}$$

where $F_{s}$ is the sampling frequency and $n_{\mathrm{DFT}}$ is the number of samples used for the DFT. The highest meaningful frequency from the DFT is $\frac{F_{s}}{2}$ [50,51], and a 0 Hz frequency (direct current) is not relevant in this work. Therefore, the used frequency range is given by Equation (4).

(4) $$\begin{array}{c} {}[\frac{F_{s}}{n_{\mathrm{DFT}}}\;:\;\frac{F_{s}}{n_{\mathrm{DFT}}}\;:\lfloor \frac{n_{\mathrm{DFT}}}{2}\rfloor \;\cdot \;\frac{F_{s}}{n_{\mathrm{DFT}}}]. \end{array}$$

The IMU sampling rate is set to $F_{s}=100\;\mathrm{Hz}$, and a time window of $\tau_{\mathrm{DFT}}=170\;ms$ is used. Therefore, $n_{\mathrm{DFT}}=17$ samples are used each time for the DFT generation: the latest values of the features in the time domain, and their values from the previous $n_{\mathrm{DFT}}-1$ samples. Therefore, the used DFT frequencies are (1) 5.8824 Hz, (ii) 11.7647 Hz, (iii) 17.6471 Hz, (iv) 23.5294 Hz, (v) 29.4118 Hz, (vi) 35.2941 Hz, (vii) 41.1765 Hz, and (viii) 47.0588 Hz. The single-sided amplitude of these 8 frequencies are the features in the frequency domain for a total of 36 features: 4 in the time domain and 32 in the frequency domain. It should be noted that due to the sliding window, it is not possible to generate a DFT for the first $n_{\mathrm{DFT}}-1$ samples. In this case, the amplitudes of their frequencies are set to zero.

By analyzing the features of the training datasets of different vehicles, it can be noticed that the dominant frequencies at standstill can be associated with the vehicle motorization. For the vehicles with internal combustion engines, to the idling Revolutions Per Minute (RPM) and the number of cylinders. When the vehicles move at walking velocity, the dominant frequency can be associated mainly with the vehicle differential. Finally, when the vehicle moves faster than walking velocity, the dominant frequencies come from the live loads of the vehicle, such as road imperfections and suspension travel, among others.

As shown in Table 1, “standstill” and “motion” are the two labels used for classification. The labeling of the training dataset can be done by using other sensors as reference, by manually analyzing the IMU signals, or a combination of both. Whichever way, it is important to notice that the labeling directly affects the classification performance of the RF.

A best practice for labeling the training datasets is to include the jerks that appear at drive-off into the “motion” class. When the vehicle comes to a stop, the “standstill” class should start only once the chassis has stopped moving and there are no more jerks or rotations present in the IMU signals. In case of uncertainty, it is preferable to label a sample as “motion” rather than “standstill” because, as is shown later, false negatives can be tolerated, but false positives are unacceptable.

The algorithm initialization and the transition between states are two effects of the proposed method to keep in mind. As for the initialization, $n_{\mathrm{DFT}}-1$ samples are needed for algorithm initialization. This is because, as explained above, the DFT can be generated only once $n_{\mathrm{DFT}}$ samples from the IMU are obtained. The single-sided amplitudes for the previous samples are set to zero, which might not be representative of the vehicle state.

Regarding the transition between states, as a vehicle goes from one state to the other (motion to standstill or vice versa), it might happen that the samples used for the DFT belong to different classes. The smaller the sliding window $\tau_{\mathrm{DFT}}$, the faster all samples for the DFT fall within a single class. Otherwise, as the sliding window $\tau_{\mathrm{DFT}}$ gets bigger, more features can be used for the classification, but this also increases the lapse where samples from both classes are fed to the RF. The current size of $\tau_{\mathrm{DFT}}$ is found to be adequate for a robust standstill recognition, and is determined after testing the RF with several hours of test datasets, and by comparing the classification performance of the RF with different values for $\tau_{\mathrm{DFT}}$.

Once the features in time and frequency domain are generated and labeled, the second step to recognize a standstill, is to train the RF. The training parameters used in this work are (i) number of trees: 12, (ii) stopping criteria: minimum leaf size, and (iii) minimum leaf size: 5. These parameters provide a robust classification and can be used for a variety of land vehicles.

It should be noted that the trained RF is not specific to the vehicle used to generate the training dataset, but to all vehicles with the same configuration (chassis, engine, transmission, suspension, etc.). This means that the training occurs only once, and the trained RF can be used for as long as the vehicle configuration remains the same.

Once the RF is trained, the third step to recognize a standstill, is to use the RF for the standstill recognition on test data. For this, the 36 features required as inputs for the RF are generated as the vehicle drives. The first step to do so is to buffer $n_{\mathrm{DFT}}$ IMU samples, i.e., the most recent IMU measurements and the previous $n_{\mathrm{DFT}}-1$. The latest IMU measurements are the features in the time domain ($a_{x,\mathrm{LCP}}^{2}+a_{y,\mathrm{LCP}}^{2}$, $a_{z,\mathrm{LCP}}^{2}$, ${\dot{\theta }}_{\mathrm{roll}}^{2}+{\dot{\theta }}_{\mathrm{pitch}}^{2}$, and ${\dot{\theta }}_{\mathrm{yaw}}^{2}$), and the features in the frequency domain are generated with all $n_{\mathrm{DFT}}$ IMU buffered samples. When all 36 features (4 in the time domain and 32 in the frequency domain) are generated, they are used as input for the RF.

If a standstill is recognized, all change rates (velocities, accelerations and rotation rates) are set to zero. Only the position and orientation are carried forward from the previous time step. Otherwise, if the vehicle moves, its state is estimated as is detailed later.

### 2.2. Horizontation of IMU Measurements

The horizontation is understood as the transformation of the IMU measurements, as if the $x_{\mathrm{LCP}}\times y_{\mathrm{LCP}}$ and $x_{\mathrm{LTP}}\times y_{\mathrm{LTP}}$ planes were parallel, and the $z_{\mathrm{LCP}}$ axis had the same orientation and direction as the $z_{\mathrm{LTP}}$ axis. The horizontation is required mainly because most vehicle motion models found in the literature depict the movement of cars from a bird’s-eye perspective. Also, it is not a practical solution to physically maintain the $x_{\mathrm{LCP}}\times y_{\mathrm{LCP}}$ and $x_{\mathrm{LTP}}\times y_{\mathrm{LTP}}$ planes parallel when the vehicles are in motion. The horizontation requires tracking of the LTP axes relative to the LCP, so that the IMU measurements can be projected on the tracked LTP axes. This process varies depending on whether the vehicle is standing still or not. Both cases are detailed in what follows.

With exception of the engine vibrations, there are no live loads present in a vehicle during a standstill. Therefore, the gravitational pull *G* is the only force sensed by the IMU. This is given at standstill by

(5) $$\begin{array}{c} G=\|a_{\mathrm{LCP}}\|=\sqrt{a_{x,\mathrm{LCP}}^{2}+a_{y,\mathrm{LCP}}^{2}+a_{z,\mathrm{LCP}}^{2}}\approx 9.81\frac{m}{s^{2}}. \end{array}$$

By using the gravitational pull as reference, the roll and pitch angles are calculated as follows

(6) $$\begin{array}{c} \theta_{\mathrm{roll}}=\arccos\left(\frac{a_{y,\mathrm{LCP}}}{G}\right), \end{array}$$

(7) $$\begin{array}{c} \theta_{\mathrm{pitch}}=\arccos\left(\frac{a_{x,\mathrm{LCP}}}{G}\right). \end{array}$$

Then, the easiest manner to obtain an initial value of the yaw angle $\theta_{\mathrm{yaw}}$, is to equip the vehicle with a sensor that measures the Course Over Ground (COG), such as a SatNav receiver, and to drive the vehicle on a straight line. This is so because driving on a straight line results in $\theta_{\mathrm{yaw}}=\mathrm{COG}$.

Two rotations are necessary to transform the LTP to the LCP: one rotation around an arbitrary axis and one rotation around the $z_{\mathrm{LTP}}$ axis. The first rotation aligns the $z_{\mathrm{LCP}}$ axis with the $z_{\mathrm{LTP}}$ axis. The second rotation aligns the $x_{\mathrm{LCP}}$ axis with the $x_{\mathrm{LTP}}$ axis. The two parameters of the first rotation are the arbitrary axis $r_{h_{0}}$ and the rotation angle $\theta_{h}$. They are expressed as follows

(8) $$\begin{array}{c} \theta_{h}=\arccos\left(\frac{a_{z,\mathrm{LCP}}}{G}\right), \end{array}$$

(9) $$r_{h_{0}}=\left[\begin{array}{ccc} a_{x,\mathrm{LCP}} & a_{y,\mathrm{LCP}} & a_{z,\mathrm{LCP}} \end{array}\right]\times \left[\begin{array}{ccc} 0 & 0 & 1 \end{array}\right]=\left[\begin{array}{ccc} r_{x,h} & r_{y,h} & r_{z,h} \end{array}\right].$$

The rotation matrix $R_{\mathrm{LCP}}^{{\mathrm{LCP}}^{\prime }}$ that aligns the $z_{\mathrm{LCP}}$ axis with the $z_{\mathrm{LTP}}$ axis is then given by:

(10) $$R_{\mathrm{LCP}}^{{\mathrm{LCP}}^{\prime }}=\left[\begin{array}{ccc} \frac{r_{x,h}^{2}+\left(r_{y,h}^{2}+r_{z,h}^{2}\right)\cos\left(\theta_{h}\right)}{r_{m}} & \frac{r_{x,h}r_{y,h}r_{c}-r_{z,h}\sqrt{r_{m}}\sin\left(\theta_{h}\right)}{r_{m}} & \frac{r_{x,h}r_{z,h}r_{c}+r_{y,h}\sqrt{r_{m}}\sin\left(\theta_{h}\right)}{r_{m}}\\ \frac{r_{x,h}r_{y,h}r_{c}+r_{z,h}\sqrt{r_{m}}\sin\left(\theta_{h}\right)}{r_{m}} & \frac{r_{y,h}^{2}+\left(r_{x,h}^{2}+r_{z,h}^{2}\right)\cos\left(\theta_{h}\right)}{r_{m}} & \frac{r_{y,h}r_{z,h}r_{c}-r_{x,h}\sqrt{r_{m}}\sin\left(\theta_{h}\right)}{r_{m}}\\ \frac{r_{x,h}r_{z,h}r_{c}-r_{y,h}\sqrt{r_{m}}\sin\left(\theta_{h}\right)}{r_{m}} & \frac{r_{y,h}r_{z,h}r_{c}+r_{x,h}\sqrt{r_{m}}\sin\left(\theta_{h}\right)}{r_{m}} & \frac{r_{z,h}^{2}+\left(r_{x,h}^{2}+r_{y,h}^{2}\right)\cos\left(\theta_{h}\right)}{r_{m}} \end{array}\right],$$

(11) $$\begin{array}{c} r_{c}=1-\cos\left(\theta_{h}\right), \end{array}$$

(12) $$\begin{array}{c} r_{m}=r_{x,h}^{2}+r_{y,h}^{2}+r_{z,h}^{2}. \end{array}$$

**Figure 2:**
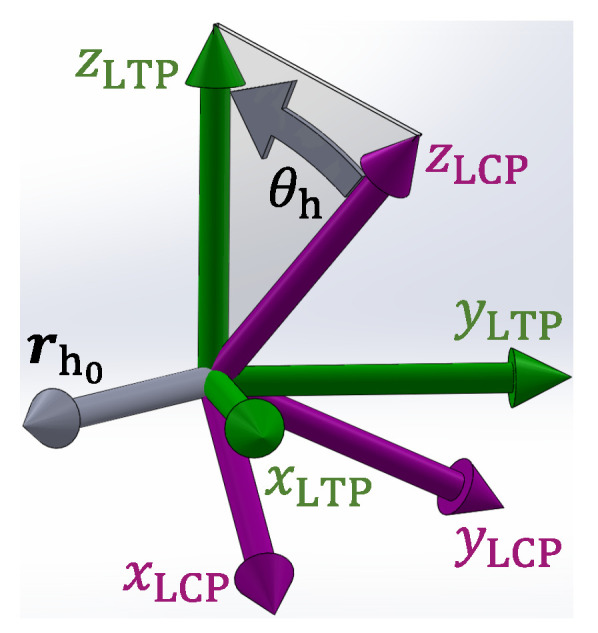
Shown is a rotation around an arbitrary axis to align the $z_{\mathrm{LCP}}$ axis with the $z_{\mathrm{LTP}}$ axis. The axes of the LTP are shown in green, the axes of the LCP are shown in magenta, and the arbitrary axis $r_{h_{0}}$ and the rotation angle $\theta_{h}$ with grey.

The Equation (10) is known as the “Rodriguez’ Formula”, and its step-by-step derivation can be found in [52]. A graphical representation of a rotation around an arbitrary axis is shown in Figure 2. It should be noted that Equation (10) can present a singularity if $\theta_{\mathrm{roll}}=0$ and $\theta_{\mathrm{pitch}}=0$, because in that case $r_{m}=0$ as well. However, this would mean that the $z_{\mathrm{LCP}}$ axis and the $z_{\mathrm{LTP}}$ axis are aligned. Hence, $R_{\mathrm{LCP}}^{{\mathrm{LCP}}^{\prime }}=I_{3\times 3}$, where I is the identity matrix.

The matrix that tracks the LTP with respect to the LCP is then given by

(13) $$R_{\mathrm{LTP}}^{\mathrm{LCP}}={\left(\left[\begin{array}{ccc} \cos\left(\theta_{\mathrm{yaw}}\right) & -\sin\left(\theta_{\mathrm{yaw}}\right) & 0\\ \sin\left(\theta_{\mathrm{yaw}}\right) & \cos\left(\theta_{\mathrm{yaw}}\right) & 0\\ 0 & 0 & 1 \end{array}\right]R_{\mathrm{LCP}}^{{\mathrm{LCP}}^{\prime }}\right)}^{T}.$$

Once the vehicle pose at standstill is determined, it must be updated as the vehicle moves. The update is done primarily with the gyroscopes because even when cruising in a straight line, random forces act on road vehicles. Some sources of these forces could be road irregularities, bumps, road joints or the wind. These forces are sensed by the IMU, which complicates the update of $R_{\mathrm{LTP}}^{\mathrm{LCP}}$ with the accelerometers. Therefore, the composition of rotations is used instead. This is detailed in what follows.

With the instantaneous rotation rates ${\dot{\theta }}_{\mathrm{roll}}$, ${\dot{\theta }}_{\mathrm{pitch}}$ and ${\dot{\theta }}_{\mathrm{yaw}}$ around the $x_{\mathrm{LCP}}$, $y_{\mathrm{LCP}}$, and $z_{\mathrm{LCP}}$ axes respectively, the instantaneous composition of rotations is expressed as follows

(14) $$S\left({\dot{\theta }}_{\mathrm{LCP},\tau_{2}}^{\mathrm{LCP},\tau_{1}}\right)=\left[\begin{array}{ccc} 0 & -{\dot{\theta }}_{\mathrm{yaw}} & {\dot{\theta }}_{\mathrm{pitch}}\\ {\dot{\theta }}_{\mathrm{yaw}} & 0 & -{\dot{\theta }}_{\mathrm{roll}}\\ -{\dot{\theta }}_{\mathrm{pitch}} & {\dot{\theta }}_{\mathrm{roll}} & 0 \end{array}\right],$$

(15) $${\dot{\theta }}_{\mathrm{LCP},\tau_{2}}^{\mathrm{LCP},\tau_{1}}=\left[\begin{array}{ccc} {\dot{\theta }}_{\mathrm{roll}} & {\dot{\theta }}_{\mathrm{pitch}} & {\dot{\theta }}_{\mathrm{yaw}} \end{array}\right].$$

Next, the instantaneous composition of rotations is integrated as if it was a vector and added to $R_{\mathrm{LTP}}^{\mathrm{LCP}}$. The step-by-step derivation of this procedure is shown below.

(16) $$\begin{array}{c} {\hat{R}}_{\mathrm{LTP}}^{\mathrm{LCP},\tau_{2}}=R_{\mathrm{LCP},\tau_{1}}^{\mathrm{LCP},\tau_{2}}R_{\mathrm{LTP}}^{\mathrm{LCP},\tau_{1}}, \end{array}$$

(17) $$\begin{array}{c} {\hat{R}}_{\mathrm{LTP}}^{\mathrm{LCP},\tau_{2}}=\left(R_{\mathrm{LCP},\tau_{1}}^{\mathrm{LCP},\tau_{1}}+\Delta \tau {\dot{R}}_{\mathrm{LCP},\tau_{1}}^{\mathrm{LCP},\tau_{2}}\right)R_{\mathrm{LTP}}^{\mathrm{LCP},\tau_{1}}, \end{array}$$

(18) $$\begin{array}{c} {\hat{R}}_{\mathrm{LTP}}^{\mathrm{LCP},\tau_{2}}=\left(R_{\mathrm{LCP},\tau_{1}}^{\mathrm{LCP},\tau_{1}}+\Delta \tau S\left({\dot{\theta }}_{\mathrm{LCP},\tau_{1}}^{\mathrm{LCP},\tau_{2}}\right)R_{\mathrm{LCP},\tau_{1}}^{\mathrm{LCP},\tau_{2}}\right)R_{\mathrm{LTP}}^{\mathrm{LCP},\tau_{1}}, \end{array}$$

(19) $$\begin{array}{c} {\hat{R}}_{\mathrm{LTP}}^{\mathrm{LCP},\tau_{2}}=\left(R_{\mathrm{LCP},\tau_{1}}^{\mathrm{LCP},\tau_{1}}+\Delta \tau S\left(-{\dot{\theta }}_{\mathrm{LCP},\tau_{2}}^{\mathrm{LCP},\tau_{1}}\right)R_{\mathrm{LCP},\tau_{1}}^{\mathrm{LCP},\tau_{2}}\right)R_{\mathrm{LTP}}^{\mathrm{LCP},\tau_{1}}, \end{array}$$

(20) $$\begin{array}{c} {\hat{R}}_{\mathrm{LTP}}^{\mathrm{LCP},\tau_{2}}=\left(I_{3\times 3}+\Delta \tau {\left(S\left({\dot{\theta }}_{\mathrm{LCP},\tau_{2}}^{\mathrm{LCP},\tau_{1}}\right)\right)}^{T}I_{3\times 3}\right)R_{\mathrm{LTP}}^{\mathrm{LCP},\tau_{1}}, \end{array}$$

(21) $$\begin{array}{c} {\hat{R}}_{\mathrm{LTP}}^{\mathrm{LCP},\tau_{2}}=\left(\Delta \tau S{\left({\dot{\theta }}_{\mathrm{LCP},\tau_{2}}^{\mathrm{LCP},\tau_{1}}\right)}^{T}+I_{3\times 3}\right)R_{\mathrm{LTP}}^{\mathrm{LCP},\tau_{1}}, \end{array}$$

where $\Delta \tau =\tau_{2}-\tau_{1}$. Equation (21) is used each time that a new IMU measurement is available to keep updating $R_{\mathrm{LTP}}^{\mathrm{LCP}}$.

To add rotations as vectors is an approximation that is valid only for rotations that tend to zero [53]. This is because ${\hat{R}}_{\mathrm{LTP}}^{\mathrm{LCP},\tau_{2}}$ is not an orthonormal matrix. Therefore, ${\hat{R}}_{\mathrm{LTP}}^{\mathrm{LCP},\tau_{2}}$ must be adjusted to make its axes perpendicular to each other. The procedure is shown in the following.

(22) $${\hat{R}}_{\mathrm{LTP}}^{\mathrm{LCP},\tau_{2}}=\left[\begin{array}{ccc} r_{h_{1}}^{\prime } & r_{h_{2}}^{\prime } & r_{h_{3}}^{\prime } \end{array}\right],$$

(23) $$\begin{array}{c} r_{h_{3}}^{\prime \prime }=r_{h_{3}}^{\prime }, \end{array}$$

(24) $$\begin{array}{c} r_{h_{1}}^{\prime \prime }=r_{h_{2}}^{\prime }\times r_{h_{3}}^{\prime \prime }, \end{array}$$

(25) $$\begin{array}{c} r_{h_{2}}^{\prime \prime }=r_{h_{3}}^{\prime \prime }\times r_{h_{1}}^{\prime \prime }, \end{array}$$

(26) $$R_{\mathrm{LTP}}^{\mathrm{LCP},\tau_{2}}=\left[\begin{array}{ccc} \frac{r_{h_{1}}^{\prime \prime }}{∥r_{h_{1}}^{\prime \prime }∥} & \frac{r_{h_{2}}^{\prime \prime }}{∥r_{h_{2}}^{\prime \prime }∥} & \frac{r_{h_{3}}^{\prime \prime }}{∥r_{h_{3}}^{\prime \prime }∥} \end{array}\right].$$

The next step is to project on the LTP the acceleration vector $a_{\mathrm{LCP}}$ and the composition of rotations ${\dot{\theta }}_{\mathrm{LCP}}$ measured by the IMU. This is done by using the tracker matrix $R_{\mathrm{LTP}}^{\mathrm{LCP},\tau_{2}}$ as follows

(27) $$a_{\mathrm{LTP},\tau_{2}}={\left(R_{\mathrm{LTP}}^{\mathrm{LCP},\tau_{2}}\right)}^{T}a_{\mathrm{LCP}}=\left[\begin{array}{ccc} a_{x,\mathrm{LTP},\tau_{2}} & a_{y,\mathrm{LTP},\tau_{2}} & a_{z,\mathrm{LTP},\tau_{2}} \end{array}\right],$$

(28) $${\dot{\theta }}_{\mathrm{LTP},\tau_{2}}={\left(R_{\mathrm{LTP}}^{\mathrm{LCP},\tau_{2}}\right)}^{T}{\dot{\theta }}_{\mathrm{LCP}}=\left[\begin{array}{ccc} {\dot{\theta }}_{x,\mathrm{LTP},\tau_{2}} & {\dot{\theta }}_{y,\mathrm{LTP},\tau_{2}} & {\dot{\theta }}_{z,\mathrm{LTP},\tau_{2}} \end{array}\right],$$

where $a_{\mathrm{LTP},\tau_{2}}$ is the acceleration vector in LTP at time instance $\tau_{2}$, ${\dot{\theta }}_{\mathrm{LTP},\tau_{2}}$ is the composition of rotations in LTP at time instance $\tau_{2}$, $a_{x,\mathrm{LTP},\tau_{2}}$, $a_{y,\mathrm{LTP},\tau_{2}}$ and $a_{z,\mathrm{LTP},\tau_{2}}$ are the accelerations at time instance $\tau_{2}$ along the $x_{\mathrm{LTP}}$, $y_{\mathrm{LTP}}$ and $z_{\mathrm{LTP}}$ axes accordingly; and ${\dot{\theta }}_{x,\mathrm{LTP},\tau_{2}}$, ${\dot{\theta }}_{y,\mathrm{LTP},\tau_{2}}$ and ${\dot{\theta }}_{z,\mathrm{LTP},\tau_{2}}$ are the rotations at time instance $\tau_{2}$ around the $x_{\mathrm{LTP}}$, $y_{\mathrm{LTP}}$ and $z_{\mathrm{LTP}}$ axes correspondingly.

The acceleration vector and composition of rotations are then projected on the $x_{\mathrm{LTP}}\times y_{\mathrm{LTP}}$-plane. The magnitude $a_{\mathrm{OG}}$ and direction $\theta_{\mathrm{aOG}}$ of the acceleration over ground are calculated as follows

(29) $$\begin{array}{c} a_{\mathrm{OG}}=\sqrt{a_{x,\mathrm{LTP},\tau_{2}}^{2}+a_{y,\mathrm{LTP},\tau_{2}}^{2}}, \end{array}$$

(30) $$\begin{array}{c} \theta_{\mathrm{aOG}}\;=\;arctan2\left(a_{y,\mathrm{LTP},\tau_{2}},a_{x,\mathrm{LTP},\tau_{2}}\right). \end{array}$$

The magnitude ${\dot{\theta }}_{\mathrm{OG}}$ and direction $\theta_{\mathrm{rOG}}$ of the composition of rotations over ground are given by

(31) $$\begin{array}{c} {\dot{\theta }}_{\mathrm{OG}}=\sqrt{{\dot{\theta }}_{x,\mathrm{LTP},\tau_{2}}^{2}+{\dot{\theta }}_{y,\mathrm{LTP},\tau_{2}}^{2}}, \end{array}$$

(32) $$\begin{array}{c} \theta_{\mathrm{rOG}}\;=\;arctan2\left({\dot{\theta }}_{y,\mathrm{LTP},\tau_{2}},{\dot{\theta }}_{x,\mathrm{LTP},\tau_{2}}\right). \end{array}$$

Once the accelerations along and rotations around the LTP axes are known, they are projected on the projection on the $x_{\mathrm{LTP}}\times y_{\mathrm{LTP}}$-plane of the longitudinal axis of the vehicle, i.e., the acceleration vector $a_{\mathrm{LTP},\tau_{2}}$ and composition of rotations ${\dot{\theta }}_{\mathrm{LTP},\tau_{2}}$ are projected on a vector located on the $x_{\mathrm{LTP}}\times y_{\mathrm{LTP}}$-plane and with $\theta_{\mathrm{yaw}}$ orientation. Therefore,

(33) $$\left[\begin{array}{c} a_{x,h}\\ a_{y,h} \end{array}\right]=\left[\begin{array}{c} a_{\mathrm{OG}}\cos\left(\theta_{\mathrm{aOG}}-\theta_{\mathrm{yaw}}\right)\\ a_{\mathrm{OG}}\sin\left(\theta_{\mathrm{aOG}}-\theta_{\mathrm{yaw}}\right) \end{array}\right],$$

(34) $$\left[\begin{array}{c} {\dot{\theta }}_{\mathrm{roll}}\\ {\dot{\theta }}_{\mathrm{pitch}} \end{array}\right]=\left[\begin{array}{c} {\dot{\theta }}_{\mathrm{OG}}\cos\left(\theta_{\mathrm{rOG}}-\theta_{\mathrm{yaw}}\right)\\ {\dot{\theta }}_{\mathrm{OG}}\sin\left(\theta_{\mathrm{rOG}}-\theta_{\mathrm{yaw}}\right) \end{array}\right],$$

where $a_{x,h}$ and $a_{y,h}$ are called “horizontal accelerations”. The measurement vector of the Extended Kalman Filter (EKF) that is designed in this research work (Section 2.3) includes ${\dot{\theta }}_{z,\mathrm{LTP}}$, $a_{x,h}$ and $a_{y,h}$ as measurement variables. The process from Equation (14) to Equation (34) is repeated with each new inertial measurement while the vehicle is in motion to keep updating the values of the measurement vector of the EKF.

### 2.3. Statistical Filtering

The next step is to estimate the vehicle state. This is done by using a motion model to predict the vehicle motion and by using exteroceptive sensors to get feedback.

If one assumes Gauss-Markov stochastic processes, the most computationally efficient method to estimate the *optimum* state of moving objects is the Kalman Filter. The Kalman Filter fuses the predicted vehicle motion with observed measurements. Its detailed derivation can be found in [15], and the specifics for the present research work are given in what follows.

The measurement vector is defined in the present work as

(35) $$z_{h}={\left[\begin{array}{cccccccc} x_{h} & y_{h} & \theta_{z,\mathrm{LTP}} & {\dot{\theta }}_{z,\mathrm{LTP}} & v_{h} & \beta_{h} & a_{x,h} & a_{y,h} \end{array}\right]}^{T},$$

where $\left(x_{h},y_{h}\right)$ are the $\left(x,y\right)$ coordinates of $o_{\mathrm{LCP}}$ in LTP, $\theta_{z,\mathrm{LTP}}$ is the yaw angle of the vehicle, $v_{h}$ is the velocity over ground of the vehicle, and $\beta_{h}$ is the sideslip angle of the vehicle. The used measurement noise covariance matrix $\sigma_{z}$ and state vector $x_{s}$ are defined as follows

(36) $$\sigma_{z}=\mathrm{diag}\left(\begin{array}{cccccccc} \sigma_{x,h}^{2} & \sigma_{y,h}^{2} & \sigma_{\theta_{z},\mathrm{LTP}}^{2} & \sigma_{{\dot{\theta }}_{z},\mathrm{LTP}}^{2} & \sigma_{v,h}^{2} & \sigma_{\beta ,h}^{2} & \sigma_{a_{x},h}^{2} & \sigma_{a_{y},h}^{2} \end{array}\right),$$

(37) $$x_{s}={\left[\begin{array}{cccccccccc} x_{s} & y_{s} & \theta_{\mathrm{yaw},s} & {\dot{\theta }}_{\mathrm{yaw},s} & v_{s} & \beta_{\mathrm{sv}} & {\dot{\beta }}_{\mathrm{sv}} & \beta_{\mathrm{sp}} & a_{x,\mathrm{sh}} & a_{y,\mathrm{sh}} \end{array}\right]}^{T},$$

where $\left(x_{s},y_{s}\right)$ are the $\left(x,y\right)$ coordinates of $o_{\mathrm{LCP}}$ in LTP, $\theta_{\mathrm{yaw},s}$ is the yaw angle, ${\dot{\theta }}_{\mathrm{yaw},s}$ is the yaw rate, $v_{s}$ is the velocity over ground, $\beta_{\mathrm{sv}}$ is the first estimation of the vehicle sideslip angle, ${\dot{\beta }}_{\mathrm{sv}}$ is the rate of change of the vehicle sideslip angle, $\beta_{\mathrm{sp}}$ is the second estimation of the vehicle sideslip angle, and $a_{x,\mathrm{sh}}$ and $a_{y,\mathrm{sh}}$ are the vehicle horizontal accelerations. Two sideslip estimators are used to improve the prediction of the vehicle motion. The first sideslip estimator ($\beta_{\mathrm{sv}}$ and ${\dot{\beta }}_{\mathrm{sv}}$) is based on the balance of moments of inertia and provides a better performance for the estimation of the vehicle velocity. The second sideslip estimator ($\beta_{\mathrm{sp}}$) is based on a geometrical model under the assumption of tractive driving, and gives a better performance for the position estimation.

With a distance $l_{r}$ along the longitudinal axis of the vehicle from $o_{\mathrm{LCP}}$ to the rear transaxle, and additive system noise $\eta_{s}$, the used state equations are given by

(38) $$\begin{array}{c} x_{\tau_{i+1},s}=f\left(x_{\tau_{i},s}\right)+\eta_{s}, \end{array}$$

(39) $$f_{{}_{\begin{array}{c} 1-10 \end{array}}}\left(x_{\tau_{i},s}\right)=\left[\begin{array}{c} x_{s}\;+\;v_{s}c\left(\theta_{\mathrm{yaw},s}\;+\;\beta_{\mathrm{sp}}\right)\Delta \tau \;+\;\left(a_{x,\mathrm{sh}}c\left(\theta_{\mathrm{yaw},s}\right)\;-\;a_{y,\mathrm{sh}}s\left(\theta_{\mathrm{yaw},s}\right)\right)\frac{\Delta \tau^{2}}{2}\\ y_{s}\;+\;v_{s}s\left(\theta_{\mathrm{yaw},s}\;+\;\beta_{\mathrm{sp}}\right)\Delta \tau \;+\;\left(a_{x,\mathrm{sh}}s\left(\theta_{\mathrm{yaw},s}\right)\;+\;a_{y,\mathrm{sh}}c\left(\theta_{\mathrm{yaw},s}\right)\right)\frac{\Delta \tau^{2}}{2}\\ \theta_{\mathrm{yaw},s}+{\dot{\theta }}_{\mathrm{yaw},s}\Delta \tau \\ {\dot{\theta }}_{\mathrm{yaw},s}\\ v_{s}+\left(a_{x,\mathrm{sh}}c\left(\beta_{\mathrm{sv}}\right)+a_{y,\mathrm{sh}}s\left(\beta_{\mathrm{sv}}\right)\right)\Delta \tau \\ \beta_{\mathrm{sv}}+{\dot{\beta }}_{\mathrm{sv}}\Delta \tau \\ \frac{1}{v_{s}}\left(a_{y,\mathrm{sh}}c\left(\beta_{\mathrm{sv}}\right)-a_{x,\mathrm{sh}}s\left(\beta_{\mathrm{sv}}\right)\right)-{\dot{\theta }}_{\mathrm{yaw},s}\\ \tan^{-1}\left(\frac{l_{r}{\dot{\theta }}_{\mathrm{yaw},s}}{v_{s}}\right)\\ a_{x,\mathrm{sh}}\\ a_{y,\mathrm{sh}} \end{array}\right].$$

From $f_{7}\left(x_{s}\right)$ and $f_{8}\left(x_{s}\right)$, one deducts that as the vehicle drives slower than $1.5\frac{m}{s}$, the sideslip estimators lead to a mathematical indetermination. To solve this, when $v_{s}<1.5\frac{m}{s}$, then $x_{s}\left(6\right)$, $x_{s}\left(7\right)$ and $x_{s}\left(8\right)$ are set to zero. Therefore, no sideslip is considered for the velocity or position estimation. However, given the low velocities where the indetermination happens, the integration error is negligible.

### 2.4. Drift Recognition

The geometrical model from which $f_{8}\left(x_{s}\right)$ is derived, assumes tractive driving. Hence, the performance of the motion model to predict the vehicle motion decays during a drift. By recognizing a drift, it is possible to (i) identify when the performance of the motion model decays, and (ii) take measures such as adjusting the system noise, or switching to other motion models. Therefore, an objective of this research work is to create a drift detector. Other approaches achieve this with multiple SatNav receivers or with specific sensors, such as the Correvit. A drift is recognized in this work by checking the consistency between state variables to reduce the dependence of INSs on external sensors.

The tire longitudinal and lateral slips are a consequence of the forces that act on the tires as well as their mechanical properties. As such, both slips indicate how much force can the tires transmit to the ground. When classified by the tire slips, the driving state of a vehicle can be classified as either “tractive” or “non-tractive” [54]. The tractive driving is characterized by high lateral and longitudinal tire tractive forces and by tire slips usually less than 10${}^{\circ }$ sideslip and less than 10% longitudinal slip. The non-tractive driving is characterized by low tire tractive forces and by high slips that are usually greater than 10${}^{\circ }$ sideslip and greater than 10% longitudinal slip.

Hence, the first pair of state variables to compare to detect a drift, are both sideslip estimators. During tractive driving, $\beta_{\mathrm{sv}}\approx \beta_{\mathrm{sp}}$. However, as the vehicle starts drifting, the difference between both gets bigger because $\beta_{\mathrm{sp}}$ is derived under the assumption of tractive driving. Therefore, the difference between $\beta_{\mathrm{sv}}$ and $\beta_{\mathrm{sp}}$ is a strong indicator that shows whether the vehicle is drifting or not.

The second pair of variables that are used for the drift recognition are the estimated lateral acceleration $a_{y,\mathrm{sh}}$ and the expected lateral acceleration $a_{y,\mathrm{sc}}$. The latter results from assuming a tire sideslip of $\beta_{t}\approx 0$. This would mean that the path of a turning vehicle describes an arch with constant radius, so that $a_{y,\mathrm{sc}}={\dot{\theta }}_{\mathrm{yaw},s}v_{s}$. Therefore, $a_{y,\mathrm{sh}}$ estimates the lateral acceleration in reality, and $a_{y,\mathrm{sc}}$ describes how the lateral acceleration should ideally be. During tractive driving, $a_{y,\mathrm{sh}}\approx a_{y,\mathrm{sc}}$. And as the vehicle starts drifting, the difference between $a_{y,\mathrm{sh}}$ and $a_{y,\mathrm{sc}}$ becomes statistically significant.

In the present work, it is said that the vehicle is drifting if $\left|a_{y,\mathrm{sh}}-a_{y,\mathrm{sc}}\right|\geq 2.1\frac{m}{s^{2}}$ and $\left|\beta_{\mathrm{sv}}-\beta_{\mathrm{sp}}\right|\geq 0.2\mathrm{rad}$. These thresholds are determined after comparing the corresponding state variables as measured during drifting tests, and as estimated during tractive driving on open roads.

### 2.5. Outlier Detection

The next step is to fuse the predicted vehicle motion with measurements from external sensors (correction data). However, the sensor information could be corrupted, which would affect negatively the estimation of the vehicle state. To avoid this, the state vector is used as reference to filter out flawed sensor measurements. This is because, by definition, the state vector represents the last known optimal state of the vehicle, and because, assuming that both the motion model and the sensors depict reality with 100% fidelity, the measurement vector is equal to the predicted vehicle state.

The consistency check is done with two consecutive sensor measurements made at time instances $\tau_{1}$ and $\tau_{2}$, which are compared to the corresponding predicted state variable. Therefore, the difference between two consecutive velocity measurements could be expressed as follows

(40) $$\begin{array}{c} v_{\tau_{2},h}-v_{\tau_{1},h}=\left(a_{x,\mathrm{sh}}c\left(\beta_{\mathrm{sv}}\right)+a_{y,\mathrm{sh}}s\left(\beta_{\mathrm{sv}}\right)\right)\Delta \tau , \end{array}$$

and to consider the inaccuracies of the sensor and the motion model, this can be rewritten as:

(41) $$\begin{array}{c} v_{\tau_{2},h}-v_{\tau_{1},h}\approx \left(a_{x,\mathrm{sh}}c\left(\beta_{\mathrm{sv}}\right)+a_{y,\mathrm{sh}}s\left(\beta_{\mathrm{sv}}\right)\right)\Delta \tau , \end{array}$$

Since the purpose is to filter outliers, an additional margin is given by means of a factor κ=3. Also, only the absolute difference is taken to consider both positive and negative accelerations of the vehicle. Therefore, the measured velocity $v_{\tau_{2},h}$ at time instance $\tau_{2}$ is used as correction data if

(42) $$\begin{array}{c} \left|v_{\tau_{2},h}-v_{\tau_{1},h}\right|\leq \left|\left(a_{x,\mathrm{sh}}c\left(\beta_{\mathrm{sv}}\right)+a_{y,\mathrm{sh}}s\left(\beta_{\mathrm{sv}}\right)\right)\Delta \tau \kappa \right|. \end{array}$$

The value of κ is determined after analyzing hours of real-world data, where the behavior of the SatNav measurements during regular operation is compared to the behavior of the measurements during periods of time where the multi-path effect is present.

Since $\theta_{\mathrm{yaw}}\approx \mathrm{COG}$ during tractive driving, the COG measured by the SatNav receivers can be used as correction data for $\theta_{\mathrm{yaw},s}$ when ${\dot{\theta }}_{\mathrm{yaw},s}\approx 0$. Analogue to the velocity measurement, the measured COG $\theta_{\tau_{2},\mathrm{yaw},h}$ at time instance $\tau_{2}$ is used as correction data if

(43) $$\begin{array}{c} \left|\theta_{\tau_{2},\mathrm{yaw},h}-\theta_{\tau_{1},\mathrm{yaw},h}\right|\leq \left|{\dot{\theta }}_{\mathrm{yaw},s}\Delta \tau \kappa \right|. \end{array}$$

Analogue to the velocity and the COG, the SatNav location measurement $\left(x_{\tau_{2},h},y_{\tau_{2},h}\right)$ at time instance $\tau_{2}$ is used as correction data if

(44) $$\begin{array}{c} \sqrt{{\left(x_{\tau_{2},h}-x_{\tau_{1},h}\right)}^{2}+{\left(y_{\tau_{2},h}-y_{\tau_{1},h}\right)}^{2}}\leq \left|\left(v_{s}+\left(a_{x,\mathrm{sh}}c\left(\beta_{\mathrm{sv}}\right)+a_{y,\mathrm{sh}}s\left(\beta_{\mathrm{sv}}\right)\right)\Delta \tau \right)\Delta \tau \kappa \right|. \end{array}$$

The same method to detect outliers can be applied for $\beta_{\mathrm{sv}}$ and $\beta_{\mathrm{sp}}$ if necessary.

Some sensors can compute quality indicators for the own measurements, such as a dilution of precision or a standard deviation. The problem is that sometimes the sensors do not recognize when they miscalculate their quality measures, as is the case with the standard deviation of SatNav receivers.

To address this, the quality measure is modelled as a PT1 element to dampen it over time. Therefore, let $\sigma_{\tau_{1},h}$ and $\sigma_{\tau_{2},h}$ be an element of $\sigma_{z}$ at time instances $\tau_{1}$ and $\tau_{2}$, $\sigma_{\tau_{1},h}^{\prime }$ and $\sigma_{\tau_{2},h}^{\prime }$ be the values corresponding to $\sigma_{\tau_{1},h}$ and $\sigma_{\tau_{2},h}$ that the sensor calculates, $t_{\mathrm{sat}}=1.5\;s$ a saturation parameter, $t_{\tau_{1}}$ and $t_{\tau_{2}}$ a timer at time instances $\tau_{1}$ and $\tau_{2}$. The update of the standard deviation is then modelled as follows

(45) $$t_{\tau_{2}}=\left\{\begin{array}{cc} 0, & \sigma_{\tau_{1},h}<\sigma_{\tau_{2},h}^{\prime }\\ t_{\tau_{1}}+\Delta \tau & \mathrm{otherwise} \end{array}\right.,$$

(46) $$\sigma_{\tau_{2},h}=\left\{\begin{array}{cc} \sigma_{\tau_{2},h}^{\prime }, & \sigma_{\tau_{1},h}<\sigma_{\tau_{2},h}^{\prime }\\ \sigma_{\tau_{1},h}e^{-\frac{t_{\tau_{2}}}{t_{\mathrm{sat}}}}+\sigma_{\tau_{2},h}^{\prime }\left(1-e^{-\frac{t_{\tau_{2}}}{t_{\mathrm{sat}}}}\right) & \mathrm{otherwise}. \end{array}\right.$$

The saturation parameter $t_{\mathrm{sat}}$ is optimized for a SatNav receiver by analyzing its measurements with and without the multi-path effect, and can be optimized for other sensors as required.

From Equations (45) and (46), it can be deducted that the strategy is to quickly shift the Kalman gain towards the motion model if the quality of the sensor measurements decays, and to gradually shift the gain towards the sensor if the quality of its measurements continuously improve.

### 2.6. LiDAR-Based Positioning Method

As stated above, there are various situations where no SatNav is available, but where highly precise correction data is required. A highly precise source of correction data is generated in this work by means of a Velodyne LiDAR HDL-32E [55], and the method is detailed in what follows.

The first step is to identify references or “markers” with the LiDAR. Since recognizing markers by their shape is computationally intensive, they are identified by their reflectivity. It should be noted that the point cloud resolution decreases (i) as the distance *d*_velo_ between the LiDAR and the measured object increases, and (ii) as the rotational velocity *ω*_velo_ of the LiDAR head increases. The Euclidean distance *d*_hor,velo_ between two horizontally adjacent points as a function of *d*_velo_ and *ω*_velo_ is given by

(47) $$\begin{array}{c} d_{\mathrm{hor},\mathrm{velo}}=d_{\mathrm{velo}}\;\cdot \;\sin\left(\omega_{\mathrm{velo}}\;\cdot \;\frac{360}{60}\;\cdot \;0.00004608\;ms\right), \end{array}$$

where 0.00004608 ms is the time required for one firing cycle (a single shot of all lasers for a single LiDAR azimuth). From Equation (47), the relation between $d_{\mathrm{velo}}$, $\omega_{\mathrm{velo}}$ and the point cloud resolution can be deducted. This relation can be very relevant as the vehicle velocity increases.

The chosen LiDAR can measure the NIST calibrated reflectivity [56], which ranges from 0 for a lost reflection to 255 for a reflection with no loses. This allows differentiation of highly reflective objects from the rest of the world, but not to identify the highly reflective objects individually.

Once only the points with high reflectivity remain, they are clustered using a time threshold of $\Gamma_{\mathrm{cluster}}=1\;ms$. Therefore, the points measured within $\Gamma_{\mathrm{cluster}}$ one another, are clustered together. Hence, all points that are within a clustering distance $d_{\mathrm{cluster}}$ from each other are clustered together, so that

(48) $$\begin{array}{c} d_{\mathrm{cluster}}=d_{\mathrm{velo}}\;\cdot \;\sin\left(\omega_{\mathrm{velo}}\;\cdot \;\frac{360}{60}\;\cdot \;\Gamma_{\mathrm{cluster}}\right). \end{array}$$

The value of $d_{\mathrm{cluster}}$ is important when defining $\omega_{\mathrm{velo}}$, $\Gamma_{\mathrm{cluster}}$ and the spacing between markers.

The next step is to determine a single set of (x,y) coordinates for the cluster. For this work, the motion of the vehicle is limited to the $x_{\mathrm{LTP}}\times y_{\mathrm{LTP}}$-plane. Therefore, the *i*-th cluster point $p_{i,\mathrm{velo}}$ is defined as

(49) $$p_{i,\mathrm{velo}}=\left[\begin{array}{c} x_{i,\mathrm{velo}}\\ y_{i,\mathrm{velo}} \end{array}\right]=\left[\begin{array}{c} d_{i,\mathrm{velo}}\;\cdot \;\cos\left(\theta_{i,\mathrm{velo}}\right)\\ d_{i,\mathrm{velo}}\;\cdot \;\sin\left(\theta_{i,\mathrm{velo}}\right) \end{array}\right],$$

and the *i*-th cluster of LiDAR measurements is then defined as

(50) $$P_{i,\mathrm{velo}}\in R^{2,n_{\mathrm{pts}}}=\left[\begin{array}{cccc} x_{1,\mathrm{velo}} & x_{i,\mathrm{velo}} & ... & x_{n_{\mathrm{pts}},\mathrm{velo}}\\ y_{1,\mathrm{velo}} & y_{i,\mathrm{velo}} & ... & y_{n_{\mathrm{pts}},\mathrm{velo}} \end{array}\right],$$

where $d_{i,\mathrm{velo}}$ and $\theta_{i,\mathrm{velo}}$ are respectively the range and azimuth angle of the *i*-th measured point, and $n_{\mathrm{pts}}$ is the number of points of the cluster.

The coordinates $p_{i,m}^{\prime }={\left[x_{i,m}^{\prime },y_{i,m}^{\prime }\right]}^{T}$ that express the measured position in LCP of the *i*-th cluster (marker) are then calculated by means of the mid-range arithmetic mean, so that

(51) $$p_{i,m}^{\prime }=\left[\begin{array}{c} x_{i,m}^{\prime }\\ y_{i,m}^{\prime } \end{array}\right]=\left[\begin{array}{c} \frac{\max\left(P_{i,\mathrm{velo}}\left(1,:\right)\right)+\min\left(P_{i,\mathrm{velo}}\left(1,:\right)\right)}{2}\\ \frac{\max\left(P_{i,\mathrm{velo}}\left(2,:\right)\right)+\min\left(P_{i,\mathrm{velo}}\left(2,:\right)\right)}{2} \end{array}\right].$$

The mid-range arithmetic is applied as well to $d_{\mathrm{velo}}$, $\theta_{\mathrm{velo}}$ and the timestamp of the clustered measurements. This allows one to obtain the distance $d_{i,m}$, the azimuth $\theta_{i,m}$ and the time instance at which the *i*-th marker is seen. Next, the true location in LTP of the markers must be known. This is defined for the *i*-th marker as follows

(52) $$\begin{array}{c} p_{i,\mathrm{map}}={\left[x_{i,\mathrm{map}},y_{i,\mathrm{map}}\right]}^{T}. \end{array}$$

The true location in LTP of all markers is stored in the marker map, which is assumed to be available beforehand. This map can be generated, for example, by means of a tachymeter or by stitching together LiDAR measurements of the test area. The marker map is then defined as follows

(53) $$P_{\mathrm{map}}\in R^{3,n_{\mathrm{pts}}}=\left[\begin{array}{cccc} 1 & i & ... & n_{\mathrm{pts}}\\ x_{1,\mathrm{map}} & x_{i,\mathrm{map}} & ... & x_{n_{\mathrm{pts}},\mathrm{map}}\\ y_{1,\mathrm{map}} & y_{i,\mathrm{map}} & ... & y_{n_{\mathrm{pts}},\mathrm{map}} \end{array}\right],$$

where $n_{\mathrm{pts}}$ is the total number markers, and the first row is a unique identifier for each marker.

In order to identify which marker is being measured, an approximate position $p_{\mathrm{app}}$ and an approximate orientation $\theta_{\mathrm{app}}$ of the vehicle in LTP is required. This approximate position can be, for example, $\left(x_{s},y_{s}\right)$, $\left(x_{h},y_{h}\right)$ or initialization values, and is defined as follows

(54) $$\begin{array}{c} p_{\mathrm{app}}={\left[x_{\mathrm{app}},y_{\mathrm{app}}\right]}^{T}. \end{array}$$

The positioning error $\eta_{d,\mathrm{app}}$ is then defined as follows

(55) $$\begin{array}{c} \eta_{d,\mathrm{app}}=\left|p_{\mathrm{app}}-p_{\mathrm{true}}\right|, \end{array}$$

were $p_{\mathrm{true}}={\left[x_{\mathrm{true}},y_{\mathrm{true}}\right]}^{T}$ is the true position of the vehicle in LTP. Since the location of the vehicle is tracked by means of an EKF, during tractive driving it holds that

(56) $$p_{\mathrm{true}}\approx p_{\mathrm{app}}\approx \left[\begin{array}{c} x_{s}\\ y_{s} \end{array}\right].$$

The approximate orientation can be $\theta_{\mathrm{yaw},s}$ or an initialization value, and during tractive driving it holds that

(57) $$\begin{array}{c} \theta_{\mathrm{true}}\approx \theta_{\mathrm{app}}\approx \theta_{\mathrm{yaw},s}, \end{array}$$

were $\theta_{\mathrm{true}}$ is the true orientation of the vehicle in LTP. The orientation error $\eta_{\theta }$ is defined as follows

(58) $$\begin{array}{c} \eta_{\theta }=\left|\theta_{\mathrm{app}}-\theta_{\mathrm{true}}\right|. \end{array}$$

So as to prevent ambiguities in the marker identification, $\eta_{d,\mathrm{app}}$ and $\eta_{\theta }$ are constrained as follows

(59) $$\begin{array}{c} \left|p_{i,m}^{\prime }\right|\sin\left(\eta_{\theta }\right)+\eta_{d,\mathrm{app}}<\frac{\min\left(d_{\mathrm{map}}\right)}{2}, \end{array}$$

where $d_{\mathrm{map}}$ is a vector with the Euclidean distance between all possible marker combinations. The markers do not have to be placed in specific patterns, as long as the constraint of Equation (59) holds. The measured position $p_{i,m}={\left[x_{i,m},y_{i,m}\right]}^{T}$ in LTP of the *i*-th marker is then given by

(60) $$p_{i,m}=p_{\mathrm{app}}+\left[\begin{array}{cc} \cos\left(\theta_{\mathrm{app}}\right) & -\sin\left(\theta_{\mathrm{app}}\right)\\ \sin\left(\theta_{\mathrm{app}}\right) & \cos\left(\theta_{\mathrm{app}}\right) \end{array}\right]p_{i,m}^{\prime }.$$

The element of $P_{\mathrm{map}}$ closest to $p_{i,m}$ is then the measured marker.

Once the markers can be individually identified, the measurement variables are calculated. The first one is the velocity over ground $v_{h}$. To calculate it, it is required that two LiDAR measurements (consecutive or not) point to the same marker. So, let $p_{\tau_{1},m}^{\prime }$ and $p_{\tau_{2},m}^{\prime }$ be the measurements in LCP of one marker done at time instances $\tau_{1}$ and $\tau_{2}$ accordingly, so that

(61) $$p_{\tau_{1},m}^{\prime }=\left[\begin{array}{c} x_{\tau_{1},m}^{\prime }\\ y_{\tau_{1},m}^{\prime } \end{array}\right]=\left[\begin{array}{c} d_{\tau_{1},m}\;\cdot \;\cos\left(\theta_{\tau_{1},m}\right)\\ d_{\tau_{1},m}\;\cdot \;\sin\left(\theta_{\tau_{1},m}\right) \end{array}\right],$$

(62) $$p_{\tau_{2},m}^{\prime }=\left[\begin{array}{c} x_{\tau_{2},m}^{\prime }\\ y_{\tau_{2},m}^{\prime } \end{array}\right]=\left[\begin{array}{c} d_{\tau_{2},m}\;\cdot \;\cos\left(\theta_{\tau_{2},m}\right)\\ d_{\tau_{2},m}\;\cdot \;\sin\left(\theta_{\tau_{2},m}\right) \end{array}\right].$$

**Figure 3:**
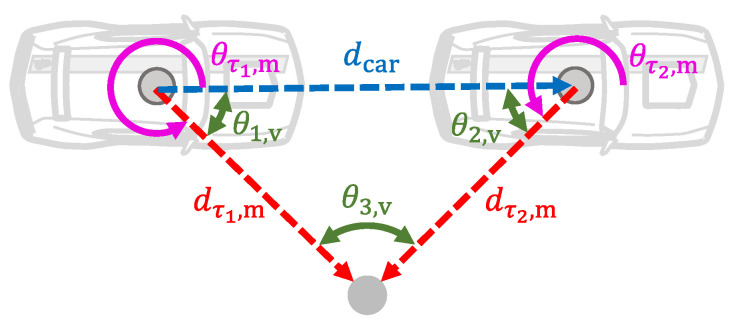
Shown is a graphical depiction of the cone geometry used for the velocity calculation from two LiDAR measurements that point to the same marker.

From $p_{\tau_{1},m}^{\prime }$ and $p_{\tau_{2},m}^{\prime }$, the geometry of a cone is constructed as shown in Figure 3.

Since the sum of the internal angles of a triangle is [π]rad, the internal angles of the constructed geometry are calculated as follows

(63) $$\theta_{1,v}=\left\{\begin{array}{cc} \theta_{\tau_{1},m}, & \theta_{\tau_{1},m}\leq \pi \\ 2\pi -\theta_{\tau_{1},m}, & \theta_{\tau_{1},m}>\pi \end{array}\right.,$$

(64) $$\theta_{2,v}=\left\{\begin{array}{cc} \pi -\theta_{\tau_{2},m}+{\dot{\theta }}_{\mathrm{yaw},s}\;\cdot \;\Delta \tau , & \theta_{\tau_{2},m}\leq \pi \\ \theta_{\tau_{2},m}+{\dot{\theta }}_{\mathrm{yaw},s}\;\cdot \;\Delta \tau -\pi , & \theta_{\tau_{2},m}>\pi \end{array}\right.,$$

(65) $$\begin{array}{c} \theta_{3,v}=\pi -\theta_{2,v}-\theta_{1,v}. \end{array}$$

The vehicle displacement $d_{\mathrm{car}}$ between the time instances $\tau_{1}$ and $\tau_{2}$ is calculated by means of the cosine law. In addition, since the LiDAR measurements have a timestamp, the measured velocity over ground of the vehicle $v_{h}$ between $\tau_{1}$ and $\tau_{2}$ is calculated as follows

(66) $$\begin{array}{c} v_{h}=\frac{d_{\mathrm{car}}}{\Delta \tau }=\frac{\sqrt{d_{\tau_{1},m}^{2}+d_{\tau_{2},m}^{2}-2\;\cdot \;d_{\tau_{1},m}\;\cdot \;d_{\tau_{2},m}\;\cdot \;\cos\left(\theta_{3,v}\right)}}{\Delta \tau }. \end{array}$$

**Figure 4:**
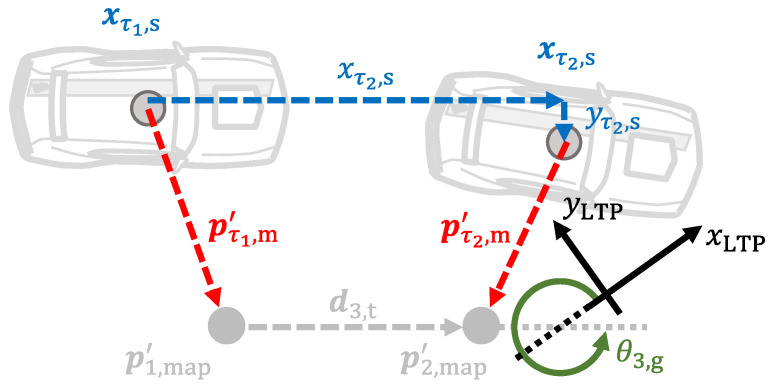
Shown is a graphical depiction of the geometry used for calculating the vehicle location.

Nevertheless, the cone geometry is not completely defined as it can rotate around the marker. Therefore, it cannot be used to calculate the vehicle location. Instead, both LiDAR measurements must point to different markers. Also, to completely define the geometry shown in Figure 4, $p_{\tau_{2},m}^{\prime }$ is expressed in the LCP at time instance $\tau_{1}$ as follows

(67) $${\tilde{p}}_{\tau_{2},m}\;=\;{\left[\begin{array}{cc} \cos\left(\theta_{\tau_{2},\mathrm{yaw},s}-\theta_{\tau_{1},\mathrm{yaw},s}\right) & -\sin\left(\theta_{\tau_{2},\mathrm{yaw},s}-\theta_{\tau_{1},\mathrm{yaw},s}\right)\\ \sin\left(\theta_{\tau_{2},\mathrm{yaw},s}-\theta_{\tau_{1},\mathrm{yaw},s}\right) & \cos\left(\theta_{\tau_{2},\mathrm{yaw},s}-\theta_{\tau_{1},\mathrm{yaw},s}\right) \end{array}\right]}^{T}\;p_{\tau_{2},m}^{\prime }\;+\;\left[\begin{array}{c} x_{\tau_{2},s}-x_{\tau_{1},s}\\ y_{\tau_{2},s}-y_{\tau_{1},s} \end{array}\right]\;=\;\left[\begin{array}{c} {\tilde{x}}_{\tau_{2},m}\\ {\tilde{y}}_{\tau_{2},m} \end{array}\right].$$

Next, assuming that $p_{\tau_{1},m}^{\prime }$ and $p_{\tau_{2},m}^{\prime }$ point to $p_{1,\mathrm{map}}$ and $p_{2,\mathrm{map}}$ respectively, the angular offset $\Xi_{\theta ,\mathrm{LCP}}$ from the LTP to the LCP at time instance $\tau_{1}$ is given by

(68) $$\begin{array}{c} \Xi_{\theta ,\mathrm{LCP}}\;=\;arctan2\left({\tilde{y}}_{\tau_{2},m}-y_{\tau_{1},m}^{\prime },{\tilde{x}}_{\tau_{2},m}-x_{\tau_{1},m}^{\prime }\right)\;-\;arctan2\left(y_{2,\mathrm{map}}-y_{1,\mathrm{map}},x_{2,\mathrm{map}}-x_{1,\mathrm{map}}\right). \end{array}$$

Afterwards, the marker measurements in LCP at time instance $\tau_{1}$ are rotated as follows

(69) $${\tilde{p}}_{\tau_{1},m}^{*}={\left[\begin{array}{cc} \cos\left(\Xi_{\theta ,\mathrm{LCP}}\right) & -\sin\left(\Xi_{\theta ,\mathrm{LCP}}\right)\\ \sin\left(\Xi_{\theta ,\mathrm{LCP}}\right) & \cos\left(\Xi_{\theta ,\mathrm{LCP}}\right) \end{array}\right]}^{T}p_{\tau_{1},m}^{\prime }=\left[\begin{array}{c} {\tilde{x}}_{\tau_{1},m}^{*}\\ {\tilde{y}}_{\tau_{1},m}^{*} \end{array}\right],$$

(70) $${\tilde{p}}_{\tau_{2},m}^{*}={\left[\begin{array}{cc} \cos\left(\Xi_{\theta ,\mathrm{LCP}}\right) & -\sin\left(\Xi_{\theta ,\mathrm{LCP}}\right)\\ \sin\left(\Xi_{\theta ,\mathrm{LCP}}\right) & \cos\left(\Xi_{\theta ,\mathrm{LCP}}\right) \end{array}\right]}^{T}{\tilde{p}}_{\tau_{2},m}=\left[\begin{array}{c} {\tilde{x}}_{\tau_{2},m}^{*}\\ {\tilde{y}}_{\tau_{2},m}^{*} \end{array}\right].$$

Finally, the linear offsets $\Xi_{\mathrm{LCP}}$ from the LTP to the LCP at time instance $\tau_{1}$ can be estimated by comparing ${\tilde{p}}_{\tau_{1},m}^{*}$ and ${\tilde{p}}_{\tau_{2},m}^{*}$ with the true marker location $p_{1,\mathrm{map}}$ and $p_{2,\mathrm{map}}$ from the marker library. Since both measurements have the same accuracy, the average of both offsets is calculated as follows

(71) $$\Xi_{\mathrm{LCP}}=\frac{\left({\tilde{p}}_{\tau_{1},m}^{*}-p_{1,\mathrm{map}}\right)+\left({\tilde{p}}_{\tau_{2},m}^{*}-p_{2,\mathrm{map}}\right)}{2}=\left[\begin{array}{c} \Xi_{x,\mathrm{LCP}}\\ \Xi_{y,\mathrm{LCP}} \end{array}\right].$$

The location and orientation measurement variables at time instances $\tau_{1}$ and $\tau_{2}$ are then given by

(72) $$\left[\begin{array}{c} x_{\tau_{1},h}\\ y_{\tau_{1},h}\\ \theta_{\tau_{1},\mathrm{yaw},h} \end{array}\right]=-\left[\begin{array}{c} \Xi_{x,\mathrm{LCP}}\\ \Xi_{y,\mathrm{LCP}}\\ \Xi_{\theta ,\mathrm{LCP}} \end{array}\right],$$

(73) $$\left[\begin{array}{c} x_{\tau_{2},h}\\ y_{\tau_{2},h}\\ \theta_{\tau_{2},\mathrm{yaw},h} \end{array}\right]=-\left[\begin{array}{c} \Xi_{x,\mathrm{LCP}}\\ \Xi_{y,\mathrm{LCP}}\\ \Xi_{\theta ,\mathrm{LCP}} \end{array}\right]+\left[\begin{array}{c} x_{\tau_{2},s}-x_{\tau_{1},s}\\ y_{\tau_{2},s}-y_{\tau_{1},s}\\ \theta_{\tau_{2},\mathrm{yaw},s}-\theta_{\tau_{1},\mathrm{yaw},s} \end{array}\right].$$

As can be deducted from the previous equations, the measurement variables for two consecutive time instances are calculated at each iteration, i.e., once the LiDAR measurement from the time instance $\tau_{2}$ is acquired, the vehicle pose for the time instances $\tau_{1}$ and $\tau_{2}$ is calculated. When the LiDAR measurement from the time instance $\tau_{3}$ is acquired, the vehicle pose for the time instances $\tau_{2}$ and $\tau_{3}$ is calculated; and so on. Since this creates an overlap of two pose measurements per time instance, both are averaged. Therefore, let $\left(x_{h,a},y_{h,a}\right)$ and $\left(x_{h,b},y_{h,b}\right)$ be the measured location of the vehicle at the *i*-th time instance that is calculated with the LiDAR measurements from the time instances i−1 to i+1. The measurement variables for the vehicle location at the *i*-th time instance are then given by

(74) $$\left[\begin{array}{c} x_{\tau_{i},h}\\ y_{\tau_{i},h} \end{array}\right]=\frac{\left[\begin{array}{c} x_{h,a}\\ y_{h,a} \end{array}\right]+\left[\begin{array}{c} x_{h,b}\\ y_{h,b} \end{array}\right]}{2}.$$

Analogously, let $\theta_{\mathrm{yaw},h,a}$ and $\theta_{\mathrm{yaw},h,b}$ be the measured orientation of the vehicle at the *i*-th time instance that is calculated with the LiDAR measurements from the time instances i−1 to i+1. The measurement variable for the vehicle orientation at the *i*-th time instance is then given by

(75) $$\begin{array}{c} \theta_{\tau_{i},\mathrm{yaw},h}=\frac{\theta_{\mathrm{yaw},h,a}+\theta_{\mathrm{yaw},h,b}}{2}. \end{array}$$

**Figure 5:**
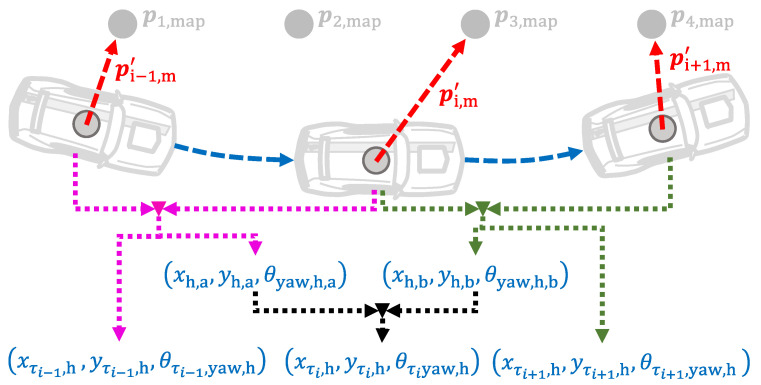
Shown is a graphical depiction of the generation of the measurement variables for the vehicle pose, as computed from LiDAR measurements.

As can be deducted from Equations (74) and (75), to average the measurement variables that are obtained using different pairs of LiDAR measurements aids to compensate LiDAR measurement errors, and creates a time-smoothing effect for the measurement vector. A graphical depiction of this process is shown in Figure 5.

To ensure that the measured object is a marker and not any other reflective object, outliers are filtered out. For this, the LiDAR measurements are translated to the LTP to compare them with the true marker position from the marker library. Therefore, the locations ${\hat{p}}_{\tau_{i},m}^{\prime }$ and ${\hat{p}}_{\tau_{i+1},m}^{\prime }$ in LTP of the LiDAR measurements $p_{\tau_{i},m}^{\prime }$ and $p_{\tau_{i+1},m}^{\prime }$ made at the *i*-th and i+1-th time instance are expressed as follows

(76) $${\hat{p}}_{\tau_{i},m}^{\prime }={\left[\begin{array}{cc} \cos\left(\Xi_{\theta ,\mathrm{LTP}}\right) & -\sin\left(\Xi_{\theta ,\mathrm{LTP}}\right)\\ \sin\left(\Xi_{\theta ,\mathrm{LTP}}\right) & \cos\left(\Xi_{\theta ,\mathrm{LTP}}\right) \end{array}\right]}^{T}p_{\tau_{i},m}^{\prime }-\Xi_{\mathrm{LTP}},$$

(77) $${\hat{p}}_{\tau_{i+1},m}^{\prime }={\left[\begin{array}{cc} \cos\left(\Xi_{\theta ,\mathrm{LTP}}\right) & -\sin\left(\Xi_{\theta ,\mathrm{LTP}}\right)\\ \sin\left(\Xi_{\theta ,\mathrm{LTP}}\right) & \cos\left(\Xi_{\theta ,\mathrm{LTP}}\right) \end{array}\right]}^{T}p_{\tau_{i+1},m}^{\prime }-\Xi_{\mathrm{LTP}}.$$

Assuming that ${\hat{p}}_{\tau_{i},m}^{\prime }$ and ${\hat{p}}_{\tau_{i+1},m}^{\prime }$ are closest to the *i*-th and i+1-th markers respectively, their measurement errors $\eta_{i,m}$ and $\eta_{i+1,m}$ are given by

(78) $$\begin{array}{c} \eta_{i,m}\;=\;\left|{\hat{p}}_{\tau_{i},m}^{\prime }-p_{i,\mathrm{map}}\right|\;=\;\eta_{i+1,m}\;=\;\left|{\hat{p}}_{\tau_{i+1},m}^{\prime }-p_{i+1,\mathrm{map}}\right|. \end{array}$$

The previous because, as shown in Equations (71) and (75), the angular and linear offsets are estimated with both LiDAR measurements.

A distance threshold is then used to separate outliers according to the following criteria:

(79) $$\left\{\begin{array}{cc} \mathrm{inlier}, & \eta_{i,m}\leq \frac{\min\left(d_{\mathrm{map}}\right)}{2}\\ \mathrm{outlier}, & \eta_{i,m}>\frac{\min\left(d_{\mathrm{map}}\right)}{2}. \end{array}\right.$$

If an outlier is detected, both measurements are discarded.

From what is detailed above, the LbPM can be used only in places with known markers, or an extra effort is needed to place and measure markers in new areas. However, traffic signs have a fixed position and are highly reflective as well. Therefore, they too can be used as markers for Simultaneous Localization and Mapping (SLAM) instead of having a marker library *a priori*.

Ongoing work of the present research focuses on evaluating the adequacy of the LbPM for SLAM purposes. A critical aspect to evaluate an LbPM-based SLAM, is to identify the sources of error individually. Since the vehicle motion prediction between LiDAR measurements can be approximated as circular movement, the LbPM does not depend on the motion model of the EKF. Hence, it is possible to analyze the motion model with no correction data and the LbPM separately. However, the recursiveness of the prediction-correction process of the EKF and localization-mapping process of the SLAM complicate the isolation of the sources of error when the EKF and the LbPM are combined.

To study separately the sources of error, the mapping performance of the LbPM-based SLAM is inspected under two circumstances: (i) with position, velocity and COG correction data; and (ii) with only velocity correction data. The first variant excludes all possible LbPM feedback errors. The second variant excludes only the velocity feedback from the LbPM as a source of error. The presented method is based on [57,58,59,60], and the specifics applicable to this work are detailed in what follows.

First, some measurement variables are considered instead as inputs to perform the prediction of vehicle state. An input vector $u_{h}$ and the control covariance matrix $Q_{k}$ are then defined as follows

(80) $$u_{h}={\left[\begin{array}{cccc} a_{x,h} & a_{y,h} & \theta_{\mathrm{yaw},h} & v_{h} \end{array}\right]}^{T},$$

(81) $$Q_{k}=diag\left(\begin{array}{cccc} \sigma_{a_{x},h}^{2} & \sigma_{a_{y},h}^{2} & \sigma_{{\dot{\theta }}_{\mathrm{yaw}}}^{2} & \sigma_{v,h}^{2} \end{array}\right).$$

With this, the vehicle state process covariance matrix can be updated as follows

(82) $$\begin{array}{c} P_{\mathrm{xx},k}=F_{x}P_{\mathrm{xx},k-1}F_{x}^{T}+F_{u}Q_{k}F_{u}^{T}, \end{array}$$

where $F_{u}$ is the Jacobian matrix of $f\left(x_{s}\right)$, derived after the control vector $u_{h}$.

Next, a measurement vector $z_{M}$ is defined as

(83) $$z_{M}={\left[\begin{array}{cc} d_{i,m} & \theta_{i,m} \end{array}\right]}^{T},$$

where $d_{i,m}$ and $\theta_{i,m}$ are respectively the range and azimuth angle of the *i*-th cluster.

The task of the observation model is to calculate the estimated measurement ${\tilde{z}}_{M}$, for a given vehicle state $x_{s}$ and one specific marker $p_{i,m}^{\prime }$. For this, two cases can be distinguished. If the current marker library is empty, then the marker is added to it with the location derived from $x_{s}$ and $z_{M}$. If the marker library is not empty, then an association check is required to compute the probability that the current observation corresponds to an existing marker. For this, the Mahalanobis Distance [61] Individual Compatibility (IC) check is performed for each marker in the library. The one yielding the minimum Mahalanobis Distance is the marker from the library with the highest probability to be the observed one. For this, the innovation term $y_{M}$ is calculated as follows

(84) $$\begin{array}{c} y_{M}=z_{M}-{\tilde{z}}_{M}. \end{array}$$

The md is then given by

(85) $$\begin{array}{c} d_{M}=\sqrt{y_{M}^{T}S_{M}^{-1}y_{M}}, \end{array}$$

with $S_{M}$ being the corresponding covariance matrix for the innovation term $y_{M}$. Finally, the marker from the library is associated with the observation according to the following criteria

(86) $$\left\{\begin{array}{cc} \mathrm{associated}, & d_{M}\leq \Gamma_{M}\\ \mathrm{not}\;\mathrm{associated}, & d_{M}>\Gamma_{M}, \end{array}\right.$$

(87) $$\begin{array}{c} \Gamma_{M}=3. \end{array}$$

This is because a threshold $\Gamma_{M}=3$ means a probability of 98.9% that the sensor measurement and the estimated measurement refer to the same marker [62].

## 3. Results

To validate the performance of the From methods that are detailed above, a series of tests are designed and performed. The From methods are tested with several hours of real-world data. In what follows, the testing details, the evaluation metrics and the most relevant results are presented.

### 3.1. Standstill Recognition

The performance of the standstill recognition is measured in terms of its classification performance according to Table 1, as well as its robustness against false positives. The four most relevant tests and their results are detailed in what follows.

**Figure 6:**
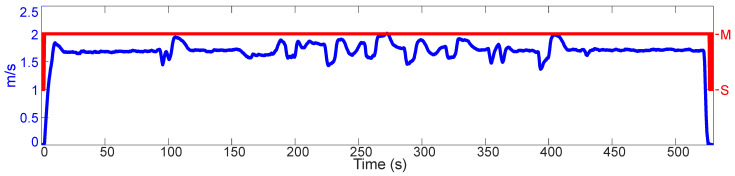
Shown is the RF output (red, S = standstill, M = motion) and the velocity over ground of the vehicle (blue). Vehicle: 3rd Gen. Smart ForTwo. Motorization: Electric Drive. Power source: Electricity.

The results of the 1st test are shown on the Figure 6. Here, a 3rd generation Smart ForTwo electric drive is placed on a test track. The INS is mounted in the trunk of the vehicle. The vehicle is turned on, the front tires are set to point straight ahead, and the gear selector placed on “D”. Some seconds after the data recording starts, the brake pedal is released. The car is driven on the test track for approximately 530 s. The accelerator pedal is not touched at any point, and the brake pedal is used only at the beginning to let the car roll and at the end to stop it. The steering wheel is used only to realign the vehicle towards paths that allow prolonged straight-line driving.

What makes this test special is the combination of (i) the low driving velocity, (ii) the drivetrain of the vehicle (electrical motor and single gear reduction transmission), and (iii) the prolonged driving moments without rotations.

As can be seen, the RF is able to recognize that the vehicle is in motion, even when driving several seconds constantly at walking velocity on a straight line: (17.87–92.53 s) and (434.80–522.00 s). It is seen as well that the RF has no false positives.

**Figure 7:**
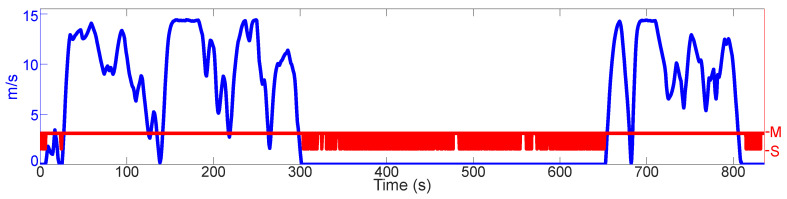
Shown is the RF output (red, S = standstill, M = motion) and the velocity over ground of the vehicle (blue). Vehicle: 5th Gen. Audi A4. Motorization: 6 cylinder, 3.0 L TDI. Power source: Diesel.

The results of the 2nd test are shown on the Figure 7. Here, a 5th generation Audi A4 3.0 L TDI with an S-Tronic 7-gear transmission is driven randomly in a city. The INS is mounted in the trunk of the vehicle. On three occasions, the gear selector is placed on “N”, and the car is allowed to roll. On one of those occasions (22.26 s), the vehicle does come to a brief stop. On the other two occasions (138.10 s and 682.20 s), the car reaches very low velocities ($0.18\frac{m}{s}$ and $0.12\frac{m}{s}$ respectively), but it does not come to a full stop. A prolonged standstill (302.10–652.00 s) is also included. During this period of time, the gear selector is kept in “S”.

This test shows (i) three rolling instances, and (ii) a prolonged standstill. Diesel engines are known to produce more vibrations than their gasoline counterparts. Also, when the gear selector is placed on “N”, much less vibrations are transmitted to the vehicle chassis because the transmission is not engaged, and because the engine idles. Therefore, the IMU signals when the vehicle rolls closely resemble those when the car is standing still, especially at low velocities. Contrarily, when the gear selector is on “S” instead of “D”, the vibrations transmitted to the chassis increase notably.

As can be seen, in the first rolling instance (22.26 s) the RF is able to recognize the standstill first when the vehicle does come to a stop and not before. Also, the RF can recognize that the vehicle is still in motion at the other two rolling instances (138.10 s and 682.20 s), despite the velocity over ground almost reaching zero. The RF is also mostly able to recognize that the vehicle is standing still (302.10–652.00 s), despite the increased vibrations provoked by selecting “S”. After 720.20 s of true standstill, the estimated total displacement of the vehicle is 0.008 m (8 mm) and the estimated total rotation of the vehicle is 0.011 rad (0.6282 deg). It is seen as well that the RF has no false positives.

**Figure 8:**
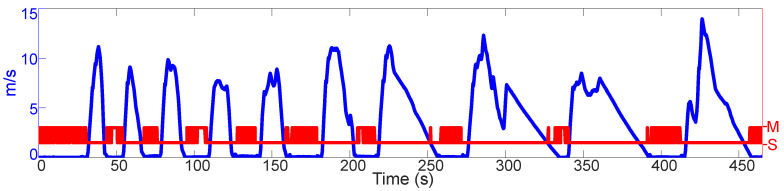
Shown is the RF output (red, S = standstill, M = motion) and the velocity over ground of the vehicle (blue). Vehicle: Suzuki GSX-R750 K2. Motorization: 4 cylinder, 749 cc. Power source: Gasoline.

The results of the 3rd test are shown on the Figure 8. Here, a Suzuki GSX-R750 K2 is driven on a test track. The INS is mounted by means of a metal plate directly on the chassis of the vehicle. On four occasions (251.80 s, 327.60 s, 391.20 s and 457.10 s), the “N” gear is selected, and the vehicle can roll to a full stop. Various instances of several seconds of standstill are included as well. In two of those instances (43.88–53.75 s and 94.08–108.20 s), the vehicle was let to rest on its side stand, with the motor idling and the gear “N” selected.

What makes this test special are (i) the chassis-drivetrain configuration, and (ii) the rolling instances. By design, the engine mounts of motorcycles are not designed to dampen the engine vibrations as well as the engine mounts of cars. Thus, these vibrations are transmitted in a much more direct manner to the motorcycle chassis. Also, given that the INS is mounted on the chassis with no dampening, it senses the engine vibrations in a much more direct manner. Thus, the INS signals under these conditions, in combination with the rolling instances, echo the signals that are present at standstill.

As can be seen, on two of the occasions where the motorcycle can roll (251.80 s and 327.60 s), false positives appear. On the other two occasions (391.20 s and 457.10 s), the RF detects a standstill first when the vehicle comes to a stop. It is seen as well that the RF has no false positives under regular driving conditions (driving always on gear).

**Figure 9:**
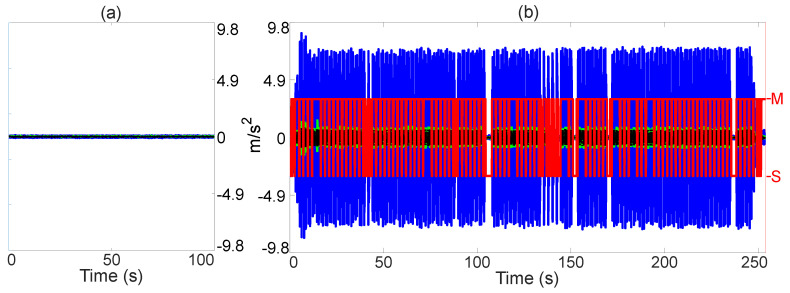
Shown are the vehicle accelerations along the $z_{\mathrm{LCP}}$ (blue), $y_{\mathrm{LCP}}$ (green) and $x_{\mathrm{LCP}}$ (black) axes. (**a**) Shows the vehicle accelerations of the RF training dataset with the label “Standstill”. (**b**) Shows the vehicle accelerations while the music is playing and the RF output (red, S = standstill, M = motion). Vehicle: 2nd Gen. Audi Q7. Motorization: 6 cylinder, 3.0 L TFSI. Power source: Gasoline.

The results of the 4th test are shown on the Figure 9. Here, a 2nd generation Audi Q7 is parked on a test track with the engine idling and the gear “P” selected. The INS is mounted in the trunk of the vehicle. The volume of the sound system is set to the maximum and music with high bass level is played for 254 s, to induce very strong vibrations for prolonged periods of time. The objective is to test if the RF can recognize the standstill regardless of the induced vibrations.

What makes this test special is the constant induction of vibrations which by magnitude is many times bigger than that of when the vehicle is standing still.

As can be seen, the output of the RF toggles much more than in the other tests. However, it is often still able to correctly detect a standstill. The maximum period of time where the vehicle state is wrongly classified as “motion” is 4.20 s, between 190.3 s and 194.50 s. In this test, after 254.01 s of true standstill, the estimated total displacement of the vehicle is 0.12 m (12.12 cm) and the estimated total rotation of the vehicle is 0.018 rad (1.08 deg).

### 3.2. Horizontation of IMU Measurements

The performance of this module is measured in terms of its capability to describe the pose (roll, pitch and yaw angles) of a vehicle. The motorcycle from the 3rd test of Section 3.1 is used because bigger roll angles can be achieved with it than with a car. The motorcycle is driven randomly on a test track, including a U-turn (772.70–780.00 s), a slalom (783.30–791.00 s), and “8” figures (791.00–843.70 s).

**Figure 10:**
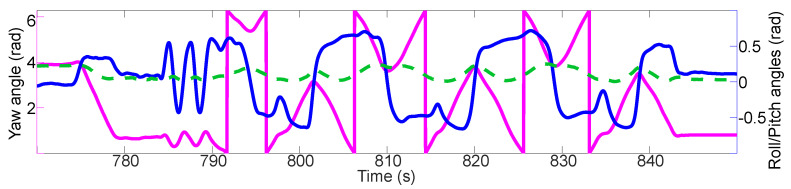
Shown are the roll (blue), pitch (green) and yaw (magenta) angles. Vehicle: Suzuki GSX-R750 K2. Motorization: 4 cylinder, 749 cc. Power source: Gasoline.

What makes this test special is that it serves as a practical demonstration of the mathematical methods detailed in Section 2.2. The results of the horizontation of the IMU measurements are shown on the Figure 10. As can be seen, the practical implementation of the mathematical methods is able to describe the vehicle pose.

### 3.3. Statistical Filtering

**Figure 11:**
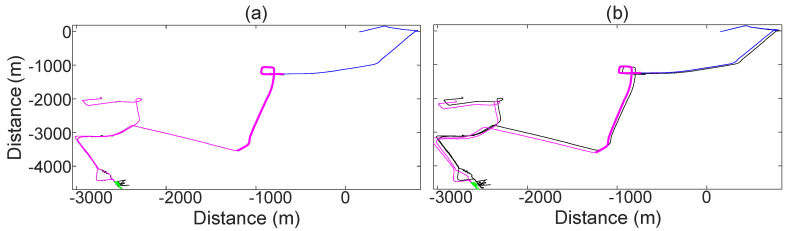
Shown is the vehicle position as measured by a SatNav receiver (black) and as estimated by means of statistical filtering (blue, magenta and green). (**a**) Shows the statistical filtering when SatNav correction data is used. (**b**) Shows the statistical filtering with no correction data.

The results of the statistical filtering are shown on the Figure 11. The performance of this module is measured in terms of its ability to (i) fuse the vehicle path (as estimated with the motion model) with correction data, (ii) estimate the vehicle path despite SatNav shortages or with corrupted SatNav measurements, and (iii) estimate the vehicle path solely by means of the motion model (with no correction data). For this, a vehicle is driven in various types of roads for 1904.15 s. The test starts at a country road (blue), followed by an express way (magenta, thick), then in-city driving (magenta, thin) and a parking structure (green). A standstill instance of 40.00 s inside the parking structure is included as well. The average velocity of the vehicle during the test, including the 40.00 s standstill inside the parking structure, is $8.19\frac{m}{s}$.

What makes this test special is (i) the inclusion of various driving conditions, (ii) the inclusion of extended periods of time without or with corrupted SatNav correction data (parking structure), and (iii) the dead-reckoning navigation.

As can be seen, the statistical filtering is able to fuse the estimation of the motion model with the SatNav measurements. It can also be seen that even in total absence of SatNav correction data, the motion model is able to accurately estimate the vehicle path. The test shown in Figure 11 is a representative case of the average accuracy of the motion model because it includes various driving situations. At the end of the test, the cumulative deviation of the position estimated by the motion model from the position measured by the SatNav receiver is 130.00 m, which accounts for a deviation of 245.79 m per driven hour at an average velocity of $8.19\frac{m}{s}$.

### 3.4. Outlier Detection

The performance of this module is measured in terms of its ability to recognize corrupted correction data, even when the sensors suggest otherwise. For this, two segments of the results of Section 3.3 are highlighted.

**Figure 12:**
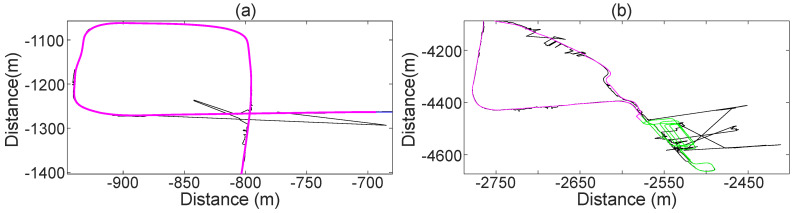
Shown is the vehicle position as measured by a SatNav receiver (black) and as estimated by means of statistical filtering (blue, magenta and green). (**a**) Shows the dataset section of a bridge underpass. (**b**) Shows the dataset section of a parking structure.

What makes this test special are the extended periods of time without or with corrupted correction data. As can be seen, the outlier detection recognizes the faulty correction data, thus dynamically adapting the Kalman gains accordingly. This greatly improves the estimation of the vehicle state. Some relevant results of the outlier detection are shown on the Figure 12.

### 3.5. Drift Recognition

**Figure 13:**
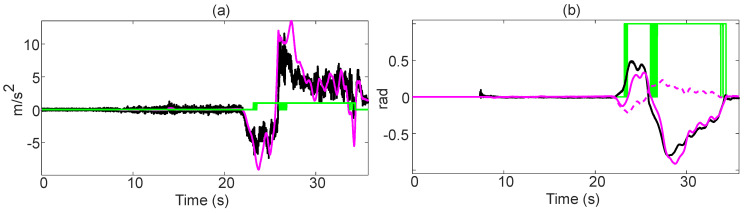
Shown are (**a**) the drift bit (green), the estimated (black) and expected (magenta) lateral acceleration, (**b**) the drift bit (green), the measured (black) and estimated (magenta solid, magenta dashed) sideslips. Drift bit = 1 means drifting.

A representative result of the drift recognition is shown on the Figure 13. The performance of this module is measured in terms of its ability to recognize that the vehicle is drifting. For this, a 3rd generation BMW M5 is driven 51 times on a test track, alternating between tractive and non-tractive driving. The test vehicle is equipped with an INS and a Correvit S-Motion. No correction data is used in these tests to test the accuracy of the motion model under these conditions.

The relevance of this test lies in (i) the extended periods of non-tractive driving, and (ii) the exclusion of correction data. As can be seen, when the vehicle enters the first curve, the module recognizes that the vehicle is not drifting. It is only when the sideslip starts to abruptly change (22.98 s) that the module starts to detect a drift. As the vehicle remains on a steady state drift (23.54–26.05 s), the drift bit stabilizes at “drifting”. During the drift transition (26.05–26.83 s), when there are moments of tractive driving, the drift bit toggles between tractive and non-tractive driving. Once the transition is finished, and the vehicle enters again into a steady state drift, the drift bit does not toggle any more. As the vehicle ends the drift (34.31 s) the drift bit toggles for a few milliseconds before stabilizing at tractive driving.

The average deviation of the final position as estimated by the motion model when compared to the position measured by the SatNav receiver for all 51 tests is ≈24.47 s, what accounts for a deviation of 2465.30 m per driven hour at $6.35\frac{m}{s}$. However, it should be noted that (i) the average duration of these tests is ≈35 s, (ii) there is tractive and non-tractive driving in each test, (iii) around the first 20 s of each test are solely straight-line driving, and (iv) there is a full reset at the beginning of every test.

### 3.6. LiDAR-Based Positioning Method

The accuracy results of the LbPM are shown on the Table 2. The performance of this module is measured in terms of its accuracy to measure the vehicle state. For this, a vehicle is equipped with an INS with RTK and a LiDAR. An array of reflective markers is placed on a test track, and the marker library is generated by measuring the markers with a SatNav receiver with RTK. Two maneuvers (a slalom and a drive-by) are driven with various velocities ranging from $5\frac{km}{h}$ and up to $40\frac{km}{h}$. The outputs from the LbPM are then compared to those of the INS with RTK. It should be noted that the INS serves as an accurate and objective reference, but no correction data is used for the LbPM.

What makes this test special are (i) the exclusion of correction data, and (ii) the validation with both maneuvers and various velocities. As can be seen, the mean accuracy of the LbPM is almost the same as that of the INS with RTK for all the state variables that the LbPM can measure.

### 3.7. LbPM Adequacy for SLAM

The adequacy of the LbPM for SLAM is measured in terms of its accuracy to generate the marker library. For this, the same test setup from Section 3.6 is used. Two tests are performed. In the first test, the position, velocity and orientation of an INS with RTK is used as correction data. In the second test, only the velocity from the INS with RTK is used as correction data. In both tests, the location of the markers is computed with the estimated vehicle state and the LiDAR measurements. The observed location of the markers is then compared to their true position from the library.

The relevance of these tests lies in that (i) it allows identify some sources of error, and (ii) it allows evaluation of the identified sources of error individually. The results are presented in Table 3. As can be seen, the marker library that is generated when the INS-RTK correction data is present, closely resembles the marker library that is generated with the RTK SatNav receiver. As expected, the marker library that is generated with only velocity correction data is less accurate. However, this inaccuracy is still smaller than that of SatNav with no correction data.

### 3.8. Runtime

The runtime results of the presented methodology are shown on Table 4. These values are obtained by executing the Matlab code of each module for 1.1+ million cycles on an Intel i7-6820HQ CPU, and by using the Matlab Profiler to measure the runtime of each module. Given that the IMU horizontation, the outlier and the drift detectors are embedded in the statistical filtering module, the runtime of these four modules is considered to be a single one.

The relevance of this test lies in that it gives a baseline to estimate the possibility of using the proposed methodology in real-time applications. As can be seen, the median runtime of all modules is always in the microsecond area. Even in a worst-case scenario (median plus 3*σ*, the runtime for the complete methodology is ≈1.33 ms, which remains under a typical IMU sampling time of 10 ms.

## 4. Discussion

As stated in Section 1, the focus of this research is to generate a reference state for ground vehicles, while reducing the dependency of the INSs on the SatNav. This allows the INSs to bridge SatNav outages for much longer periods of time, or even to function with no SatNav at all. Specifically, for the automotive industry, there are various use-cases that justify the use of SatNav-deprived INSs, such as the navigation in tunnels, underpasses or parking structures. A highly precise and prolonged navigation in places with no SatNav, such as testing halls, is also very relevant for the automotive research. As shown in Section 3, the methods developed in this research work address the aspects where the INSs profit the most.

Starting with the standstill recognition, it is shown that it is possible to classify whether a vehicle is moving or standing still by using only machine learning techniques and IMU measurements. The fact that no additional sensor is required, clearly implies important advantages, such as (i) reduced costs, (ii) less testing complexity, (iii) simplified information processing, and (iv) stand-alone functioning.

The proposed method uses the same features in the frequency domain, regardless of the vehicle. However, a first glance at the Laplace transformation of the features in the time domain, suggests that the choice of frequencies can be further refined if one considers them on a vehicle-specific basis. This could eventually lead to an improvement in the standstill recognition. Therefore, future work could include a vehicle-specific analysis of the features in the frequency domain. The use of the on-board vehicle sensors could also help refine and automate the data labeling.

As for the horizontation of INS measurements, modern INSs do use correction data (typically from the SatNav) to refine the IMU measurements. However, it is shown that the pose of the vehicle can be accurately described with the raw IMU measurements. This implies independence from the SatNav. The inclusion of the detailed mathematics to horizontate IMU measurements, implies an aid to scientists working on similar topics or trying to replicate the results presented here.

One challenging aspect of the horizontation of the IMU measurements is to detect the IMU mounting pose in the vehicle, i.e., it is possible to estimate the pose of the IMU with respect to the gravity vector and the true north of the Earth. However, it is not an easy task to relate the pitch and roll of the IMU with that of the vehicle because the IMU could be mounted in such a manner that its x×y-plane is not parallel to the x×y-plane of the vehicle. Knowing the offsets between both planes could be highly valuable to recognize certain driving situations, such as driving inside parking structures, wheelies or stoppies. Since the acquisition of these offsets on a vehicle-to-vehicle basis is a complicated and time-consuming task, future work could include the investigation of automated methods that allow estimation of these offsets.

The KF is a computationally efficient algorithm for fusing data. However, the accuracy of its output is limited by the accuracy of the information to fuse. Therefore, it is important to refine the sources of information as much as possible before using them as inputs for the KF. In this work, the EKF fuses the state vector estimated by the motion model with the measurements of the vehicle state made with external sensors. Therefore, a lot of effort is put on refining the motion model for tractive-driving applications. As shown above, the proposed motion model can accurately estimate the vehicle state for extended periods of time with no correction data, which implies a high confidence on the vehicle state, even when navigating by dead reckoning.

Given that the motion model is designed to perform best for tractive driving, its performance is reduced during non-tractive driving. Also, given that the drifting tests are designed to test the drift recognition, they are not long enough to objectively determine the accuracy of the motion model during a drift. Therefore, future work could include a deeper investigation of the accuracy of the motion model during non-tractive driving. This could help to estimate adequate values for the system noise, and so improve the performance of the KF during drifting.

As for the outlier detector, it further helps in refining the sources of information before they are fed to the KF. Its effect is especially noticeable when the sensors deliver quality metrics for their own measurements, as is the case of the standard deviation for the SatNav. As shown above, to include a smart outlier detector implies a very useful consistency check of the sensor measurements, thus avoiding fusing information that would negatively affect the estimation of the vehicle state.

This detector uses the latest state vector because it is the last known vehicle state. However, as time passes, the predicted state without correction data might differ so much from the true state that the constraints shown in Section 2.5 filter out meaningful sensor measurements. Future work could include the combination of the system noise with the state vector to better adapt the outlier detector.

Yet another step that is taken to improve the KF is the drift detector. Given that the motion model is designed to function best in tractive driving, it is very useful to differentiate whether the vehicle is drifting or not. As shown above, the drift recognition can make this distinction. This implies that the time instance to make changes in the KF is known. This can be, for example, to adjust the system noise accordingly or to use a more adequate motion model.

The drift recognition here presented is based on the thresholds of two features: the magnitude of the sideslip and the difference between the estimated and expected lateral acceleration. Future work on this method could include the analysis of a bigger dataset that includes vehicles with different drivetrains (front, rear and all-wheel drive). This could help to gain a better understanding on the ideal thresholds for vehicles according to their drivetrain.

One crucial module to reduce the dependency of INSs on the SatNav is the LbPM. The results clearly show that the accuracy of the vehicle state as measured by this method closely resembles that of an INS with RTK correction data. Considering the refresh rate of the LiDAR and the state variables that can be measured by means of the LbPM, this could already be enough for an accurate vehicle indoor navigation. Future work on this module could include the implementation of the method on dedicated hardware to analyze the computing requirements for a real-time implementation.

The results on Table 3, suggest that the LbPM is adequate for SLAM. Given that the LbPM is capable of delivering RTK-like correction data, it could enable the generation of an accurate marker library in unknown environments. It is not clear whether or not a lane-accurate indoor navigation could be possible in places with a reduced marker density. Future work on this module could include adapting the LbPM algorithm for SLAM, and implementing it on dedicated hardware for real-world testing. For this purpose, tailored-made hardware solutions, such as [63,64], could aid in the navigation of complex environments, as can be parking structures. The use of lightweight artificial intelligence techniques, as shown in [65,66] could aid in the implementation of the standstill recognition in hardware with limited computational resources, as are embedded systems. Finally, the sensor fusion with on-board sensors in the vehicles could improve the dead-reckoning navigation in indoor environments.

Regarding the methodology runtime, it should be noted that even with high-level programming code, such as a Matlab, its runtime never exceeded 2 ms. This implies that it is possible to implement the methodology in real-time applications. Future work could include the implementation using a more efficient programming language, such as C++, and the use of dedicated hardware.

## 5. Conclusions

The objective of generating a reference vehicle state for proper testing and validation of automated driving functions is achieved in a prototypical manner by developing novel methods and by adapting existing ones to the problems at hand. This while reducing the dependency of INSs on external sensors, such as the SatNav. The testing results demonstrate that the proposed methods greatly improve the accuracy of the estimated vehicle state. The measured runtime suggests the possibility of real-time implementation of the proposed methodology.

## Figures and Tables

**Table 1:** Standstill classification.

	Classified State	Standstill	Motion
True State			
Standstill		True positive	False negative
Motion		False positive	True negative

**Table 2:** Accuracy results for the proposed LbPM. Shown are the maneuvers, maneuverer velocity, mean deviation from the reference and Std. dev. of the corresponding errors.

Manoeuvrers	Positioning Accuracy		Orientation Accuracy		Velocity Accuracy	
**Drive-by**	**Mean (m)**	**Std. dev. (m)**	**Mean (deg)**	**Std. dev.** **(deg)**	**Mean** $\left(\frac{m}{s}\right)$	**Std. dev.** $\left(\frac{m}{s}\right)$
5 $\frac{km}{h}$	0.04	0.02	0.73	0.25	0.06	0.08
10 $\frac{km}{h}$	0.03	0.02	0.19	0.20	0.08	0.10
15 $\frac{km}{h}$	0.03	0.02	0.26	0.19	0.07	0.09
20 $\frac{km}{h}$	0.03	0.02	0.37	0.23	0.08	0.09
25 $\frac{km}{h}$	0.04	0.02	0.58	0.23	0.08	0.10
30 $\frac{km}{h}$	0.06	0.02	0.51	0.22	0.08	0.10
35 $\frac{km}{h}$	0.07	0.03	0.44	0.25	0.08	0.11
40 $\frac{km}{h}$	0.08	0.03	0.41	0.26	0.11	0.13
**Slalom**	**Mean (m)**	**Std. dev. (m)**	**Mean (deg)**	**Std. dev.** **(deg)**	**Mean** $\left(\frac{m}{s}\right)$	**Std. dev.** $\left(\frac{m}{s}\right)$
5 $\frac{km}{h}$	0.04	0.02	0.24	0.29	0.08	0.11
10 $\frac{km}{h}$	0.04	0.02	0.40	0.29	0.09	0.12
20 $\frac{km}{h}$	0.04	0.02	0.32	0.36	0.14	0.17
30 $\frac{km}{h}$	0.05	0.02	0.36	0.40	0.18	0.24
40 $\frac{km}{h}$	0.10	0.02	0.53	0.43	0.18	0.22

**Table 3:** Results of the mapping accuracy. Shown are the maneuvers, driving velocity, mean deviation from the observed to the true marker location, std. dev. of the errors and the maximum deviation. The columns (**a**) show the results when using position, velocity and orientation correction data. The columns (**b**) show the results when using only velocity correction data.

Manoeuvre	Mapping Accuracy			Manoeuvre	Mapping Accuracy		
Drive-by	Mean (m)	Std. dev. (m)	Max (m)	Slalom	Mean (m)	Std. dev. (m)	Max (m)
	(a)/(b)	(a)/(b)	(a)/(b)		(a)/(b)	(a)/(b)	(a)/(b)
5 $\frac{km}{h}$	0.11/0.90	0.04/0.51	0.19/1.56	5 $\frac{km}{h}$	0.33/0.93	0.24/0.51	0.81/1.62
10 $\frac{km}{h}$	0.11/0.13	0.04/0.06	0.19/0.22	10 $\frac{km}{h}$	0.08/0.27	0.03/0.11	0.14/0.42
15 $\frac{km}{h}$	0.12/0.18	0.05/0.07	0.22/0.34	15 $\frac{km}{h}$	0.08/0.53	0.03/0.25	0.16/1.04
20 $\frac{km}{h}$	0.14/0.29	0.06/0.12	0.28/0.48	20 $\frac{km}{h}$	0.08/0.78	0.03/0.56	0.14/1.70
25 $\frac{km}{h}$	0.17/0.11	0.08/0.06	0.32/0.39	25 $\frac{km}{h}$	0.10/0.30	0.05/0.14	0.23/0.64
30 $\frac{km}{h}$	0.14/0.16	0.06/0.09	0.28/0.44	30 $\frac{km}{h}$	0.08/0.67	0.04/0.36	0.21/1.53
35 $\frac{km}{h}$	0.10/0.20	0.05/0.15	0.20/0.69	35 $\frac{km}{h}$	0.11/0.96	0.06/0.52	0.25/1.96
40 $\frac{km}{h}$	0.10/0.38	0.05/0.19	0.23/0.77	40 $\frac{km}{h}$	0.11/0.26	0.06/0.15	0.25/0.64

**Table 4:** Shown is the median and the std. dev. of the runtime of the modules presented above.

Module	Median (μs)	Std. dev. (μs)
Standstill classifier	123	50
Statistical filtering	403	98
LbPM-point clustering	43	20
LbPM-velocity estimation	11	14
LbPM-position estimation	42	54

## Data Availability

Datasets of the IMU signals and SatNav measurements of the results shown are available within the article as Supplementary Materials. The data presented in this study are available within the article as Supplementary Materials.

## Supplementary Materials

The following are available online at https://www.mdpi.com/1424-8220/21/4/1131/s1.

## Abbreviations

The following abbreviations are used in this manuscript:

SatNav	Satellite Navigation
IMU(s)	Inertial Measurement Unit(s)
INS(s)	Inertial Navigation System(s)
RTK	Real-Time Kinematic
LbPM	LiDAR-based Positioning Method
RF	Random Forest
DFT	Discrete Fourier Transform
RPM	Revolutions Per Minute
COG	Course Over Ground
EKF	Extended Kalman Filter
GPS	Global Positioning System

## References

[1] Einstein A. (1920). Relativity.

[2] Schulze O. (1902). Wirbelstrom-Tachometer. German.

[3] Tindell K., Burns A. Guaranteeing message latencies on Controller Area Network (CAN). Proceedings of the 1st International CAN Conference.

[4] Tindell K., Hanssmon H., Wellings A.J. Analysing Real-Time Communications: Controller Area Network (CAN). Proceedings of the RTSS Citeseer.

[5] Tindell K., Burns A., Wellings A.J. (1995). Calculating controller area network (CAN) message response times. Control Eng. Pract..

[6] Casparsson L., Rajnak A., Tindell K., Malmberg P. (1998). Volcano—A Revolution in On-Board Communications.

[7] DeMeis R. (2005). Cars sag under weighty wiring. EE Times.

[8] Davis R.I., Burns A., Bril R.J., Lukkien J.J. (2007). Controller Area Network (CAN) schedulability analysis: Refuted, revisited and revised. Real Time Syst..

[9] Davis R.I., Navet N. Controller area network (CAN) schedulability analysis for messages with arbitrary deadlines in FIFO and work-conserving queues. Proceedings of the 2012 9th IEEE International Workshop on Factory Communication Systems.

[10] Dziurzanski P., Davis R.I., Indrusiak L.S. Synthesizing Real-Time Schedulability Tests using Evolutionary Algorithms: A Proof of Concept. Proceedings of the 2019 IEEE Real-Time Systems Symposium (RTSS).

[11] WPT Sensors: Wheel Pulse Transducers. https://www.kistler.com/files/document/000-811e.pdf.

[12] Correvit S-Motion DTI: 2-axis Optical Sensors. https://www.kistler.com/files/document/003-395e.pdf.

[13] Haus J., Lauinger N. (2007). Optische Gitter: Die Abbildung der Realität—75 Jahre berührungslose dynamische Meßtechnik auf der Basis optischer Gitter. Laser Tech. J..

[14] (2013). Robert Bosch GMBH Vehicle Standstill Recognition. German.

[15] Kalman R. (1960). A New Approach to Linear Filtering and Prediction Problems. J. Basic Eng..

[16] Zubov I., Afanasyev I., Gabdullin A., Mustafin R., Shimchik I. Autonomous Drifting Control in 3D Car Racing Simulator. Proceedings of the 2018 International Conference on Intelligent Systems (IS).

[17] Wang H., Zhao L., Yang Y., Yan Y., Liu J. Modeling and analysis of wheel mobile robot for high-speed precise drift. Proceedings of the 31st Chinese Control Conference.

[18] Bárdos A., Domina A., Szalay Z., Tihanyi V., Palkovics L. MIMO Controller Design for Stabilizing Vehicle Drifting. Proceedings of the 2019 IEEE 19th International Symposium on Computational Intelligence and Informatics and 7th IEEE International Conference on Recent Achievements in Mechatronics, Automation, Computer Sciences and Robotics (CINTI-MACRo).

[19] El-Saigh A.I., Macario R.C.V. A review of anti-multipath techiques, past and present. Proceedings of the IEE Colloquium on Multipath Countermeasures.

[20] Burr A.G. The multipath problem: An overview. Proceedings of the IEE Colloquium on Multipath Countermeasures.

[21] Cheng L., Chen J., Gan M. Multipath error analysis of carrier Tracking Loop in GPS receiver. Proceedings of the 29th Chinese Control Conference.

[22] Liu L., Amin M.G. Comparison of Average Performance of GPS Discriminators in Multipath. Proceedings of the 2007 IEEE International Conference on Acoustics, Speech and Signal Processing-ICASSP‘07.

[23] Yedukondalu K., Sarma A.D., Kumar A. Mitigation of GPS multipath error using recursive least squares adaptive filtering. Proceedings of the 2010 IEEE Asia Pacific Conference on Circuits and Systems.

[24] Groves P.D. (2013). Principles of GNSS, Inertial, and Multisensor Integrated Navigation Systems.

[25] Farrell J. (2008). Aided Navigation: GPS with High Rate Sensors.

[26] Program Funding. https://www.gps.gov/policy/funding/.

[27] Annual Activity Report of the European GNSS Agency 2019. https://www.gsa.europa.eu/sites/default/files/annual_report_gsa_2019.pdf.

[28] New Civil Signals. https://www.gps.gov/systems/gps/modernization/civilsignals/.

[29] Release No: CR-048-17. https://www.defense.gov/News/Contracts/Contract-View/Article/1112618/.

[30] Lam K. Broadcom Introduces World’s First Dual Frequency GNSS Receiver with Centimeter Accuracy for Consumer LBS Applications. https://www.broadcom.com/company/news/product-releases/2302120.

[31] Beidou Upgrades for Global Reach. https://en.unicorecomm.com/news/detail/18.

[32] Batistić L., Tomic M. Overview of indoor positioning system technologies. Proceedings of the 2018 41st International Convention on Information and Communication Technology, Electronics and Microelectronics (MIPRO).

[33] Einsiedler J., Radusch I., Wolter K. Vehicle indoor positioning: A survey. Proceedings of the 2017 14th Workshop on Positioning, Navigation and Communications (WPNC).

[34] iBeacon-Apple Developer. https://developer.apple.com/ibeacon/.

[35] Ibisch A., Stümper S., Altinger H., Neuhausen M., Tschentscher M., Schlipsing M., Salinen J., Knoll A. Towards autonomous driving in a parking garage: Vehicle localization and tracking using environment-embedded LIDAR sensors. Proceedings of the 2013 IEEE Intelligent Vehicles Symposium (IV).

[36] Harter A., Hopper A., Steggles P., Ward A., Webster P. (2002). The Anatomy of A Context-Aware Application. Wirel. Netw..

[37] Ward A., Jones A., Hopper A. (1997). A New Location Technique for the Active Office. IEEE Pers. Commun..

[38] Addlesee M., Curwen R., Hodges S., Newman J., Steggles P., Ward A., Hopper A. (2001). Implementing a sentient computing system. Computer.

[39] Indoor Positioning System-Data Drives Innovation. https://indoo.rs/solution/indoor-positioning-system/.

[40] Quick Start: Indoor Positioning Systems. https://www.infsoft.com/indoor-positioning.

[41] What Is Indoor Positioning Systems?. https://senion.com/indoor-positioning-system/#difference.

[42] Gatesichapakorn S., Takamatsu J., Ruchanurucks M. ROS based Autonomous Mobile Robot Navigation using 2D LiDAR and RGB-D Camera. Proceedings of the 2019 First International Symposium on Instrumentation, Control, Artificial Intelligence, and Robotics (ICA-SYMP).

[43] Toth C., Grejner-Brzezinska D.A., Lee Y. Terrain-based navigation: Trajectory recovery from LiDAR data. Proceedings of the 2008 IEEE/ION Position, Location and Navigation Symposium.

[44] Cong D., Zhang L., Su P., Tang Z., Meng Y., Wang Y. Design and implementation of LiDAR navigation system based on triangulation measurement. Proceedings of the 2017 29th Chinese Control And Decision Conference (CCDC).

[45] ISO 8855:2011 (2011). ISO/TC 22/SC 33 Vehicle Dynamics and Chassis Components.

[46] Breiman L. (2001). Random forests. Mach. Learn..

[47] Frigo M., Johnson S.G. (2005). The Design and Implementation of FFTW3. Proc. IEEE.

[48] Johnson S.G., Frigo M. (2007). A modified split-radix FFT with fewer arithmetic operations. IEEE Trans. Signal Process..

[49] Frigo M., Leiserson C.E., Prokop H., Ramachandran S. Cache-oblivious algorithms. Proceedings of the 40th Annual Symposium on Foundations of Computer Science.

[50] Nyquist H. (1928). Certain Topics in Telegraph Transmission Theory. Trans. Am. Inst. Electr. Eng..

[51] Shannon C.E. (1949). Communication in the Presence of Noise. Proceedings of the 1949 Proceedings of the Institute of Radio Engineers.

[52] Murray G. Rotation about an Arbitrary Axis in 3 Dimensions. https://tinyurl.com/y6rvtbtx.

[53] Caicedo M. Cinemática de las Rotaciones. http://hc09paa1.pbworks.com/f/FisII_rotation.pdf.

[54] Abdulrahim M. (2006). On the Dynamics of Automobile Drifting. SAE Mobilus.

[55] Velodyne LiDAR, Inc. (2017). HDL-32E User Manual.

[56] Laser Measurements Calibrations. https://www.nist.gov/calibrations/laser-measurements-calibrations.

[57] Durrant-Whyte H., Bailey T. (2006). Simultaneous localization and mapping: Part I. IEEE Robot. Autom. Mag..

[58] Mochnac J., Marchevsky S., Kocan P. Bayesian filtering techniques: Kalman and extended Kalman filter basics. Proceedings of the 2009 19th International Conference Radioelektronika.

[59] Matsebe O., Namoshe M., Tlale N. (2010). Basic Extended Kalman Filter-Simultaneous Localisation and Mapping.

[60] Dissanayake M.W.M.G., Newman P., Clark S., Durrant-Whyte H.F., Csorba M. (2001). A solution to the simultaneous localization and map building (SLAM) problem. IEEE Trans. Robot. Autom..

[61] Mahalanobis P.C. (1936). On the Generalized Distance in Statistics. Proc. Natl. Inst. Sci. India.

[62] Wang B., Shi W., Miao Z. (2015). Confidence Analysis of Standard Deviational Ellipse and Its Extension into Higher Dimensional Euclidean Space. PLoS ONE.

[63] Lindner L., Sergiyenko O., Rivas-López M., Ivanov M., Rodríguez-Quiñonez J.C., Hernández-Balbuena D., Flores-Fuentes W., Tyrsa V., Muerrieta-Rico F.N., Mercorelli P. Machine vision system errors for unmanned aerial vehicle navigation. Proceedings of the 2017 IEEE 26th International Symposium on Industrial Electronics (ISIE).

[64] Garcia-Cruz X., Sergiyenko O., Tyrsa V., Rivas-Lopez M., Hernandez-Balbuena D., Rodriguez-Quiñonez J., Basaca-Preciado L., Mercorelli P. (2014). Optimization of 3D laser scanning speed by use of combined variable step. Opt. Lasers Eng..

[65] Gupta N., Khosravy M., Gupta S., Dey N., González Crespo R. (2020). Lightweight Artificial Intelligence Technology for Health Diagnosis of Agriculture Vehicles: Parallel Evolving Artificial Neural Networks by Genetic Algorithm. Int. J. Parallel Program..

[66] Gupta N., Khosravy M., Patel N., Dey N., Gupta S., Darbari H., González Crespo R. (2020). Economic data analytic AI technique on IoT edge devices for health monitoring of agriculture machines. Appl. Intell..

